# Identification of Genetic Variations and Candidate Genes Responsible for Stalk Sugar Content and Agronomic Traits in Fresh Corn via GWAS across Multiple Environments

**DOI:** 10.3390/ijms232113490

**Published:** 2022-11-04

**Authors:** Jianjian Chen, Jinming Cao, Yunlong Bian, Hui Zhang, Xiangnan Li, Zhenxing Wu, Guojin Guo, Guihua Lv

**Affiliations:** 1 Institute of Maize and Featured Upland Crops, Zhejiang Academy of Agricultural Sciences, Hangzhou 310004, China; 2 Jiangsu Key Laboratory of Crop Genomics and Molecular Breeding, Co-Innovation Center for Modern Production Technology of Grain Crops, Key Laboratory of Plant Functional Genomics of the Ministry of Education, Jiangsu Key Laboratory of Crop Genetics and Physiology, Yangzhou University, Yangzhou 225009, China; 3 Zhejiang Agricultural Technology Extension Center, Hangzhou 310004, China

**Keywords:** fresh corn, stalk sugar content, agronomic traits, genome-wide association analysis, selective sweep, single nucleotide polymorphisms

## Abstract

The stem and leaves of fresh corn plants can be used as green silage or can be converted to biofuels, and the stalk sugar content and yield directly determine the application value of fresh corn. To identify the genetic variations and candidate genes responsible for the related traits in fresh corn, the genome-wide scan and genome-wide association analysis (GWAS) were performed. A total of 32 selective regions containing 172 genes were detected between sweet and waxy corns. Using the stalk sugar content and seven other agronomic traits measured in four seasons over two years, the GWAS identified ninety-two significant single nucleotide polymorphisms (SNPs). Most importantly, seven SNPs associated with the stalk sugar content were detected across multiple environments, which could explain 13.68–17.82% of the phenotypic variation. Accessions differing in genotype for certain significant SNPs showed significant variation in the stalk sugar content and other agronomic traits, and the expression levels of six important candidate genes were significantly different between two materials with different stalk sugar content. The genetic variations and candidate genes provide valuable resources for future studies of the molecular mechanism of the stalk sugar content and establish the foundation for molecular marker-assisted breeding of fresh corn.

## 1. Introduction

The stem and leaves of fresh corn plants are often applied to green silage and converted to biofuels. Fresh corn, mainly comprising sweet corn and waxy corn types, is an important staple food consumed as a vegetable or fruit, and is also used for livestock feed and fuel [1]. The stems and leaves of fresh corn plants, after harvesting of the ears, are rich in nutrients and can be used as green silage, thus realizing the utilization of multiple corn resources [2]. Corn silage is widely used in animal husbandry because of its high energy value [3]. Therefore, improvement of the quality of silage corn feed is important for the development of animal husbandry. Fresh corn must go through a suitable fermentation process to become high-quality silage, which produces a large amount of lactic acid, and sugar is the crucial component in this process [4,5]. However, an insufficient amount of lactic acid not only leads to the reduction in silage quality but also might favor silage mildew development. Therefore, the sugar content of green silage is an important factor that affects silage quality, and a higher stalk sugar content can improve the feed quality and palatability of silage corn [6,7]. In addition, plant height, plant weight, and stalk sugar content are important factors that affect the utility of fresh corn; increasing the plant weight and stalk sugar content of fresh corn can promote the application of corn green silage and promote the development of animal husbandry. Most importantly, the stalk sugar content is also an important attribute affecting the overall feasibility and profitability of biofuel production, such as bioethanol. Given the current challenges faced in the world, identifying biomass resources with superior properties is important to balance food safety and biofuel production [8]. Increasing the stalk sugar content of fresh corn will facilitate a more economically viable production of higher-generation biofuels, which are derived from biomass rather than from edible resources.

Sugar supply is crucial for plant growth and development. Calcium signaling-mediated transcriptional regulation might play an important role in glucose metabolism. Calcium-dependent protein kinases can phosphorylate and regulate sucrose synthase and sucrose phosphate synthase, thus influencing sucrose accumulation [9,10]. Receptor protein kinases may regulate sucrose metabolism in sugarcane [11], and MYB transcription factors are involved in the regulation of water-soluble polysaccharide biosynthesis [12,13]. Pyruvate dehydrogenase plays an important role in energy production by controlling the entry of carbon into the tricarboxylic acid cycle [14,15]. Mutation of Sh2 (shrunken2), which encodes the large subunit of adenosine diphosphate glucose pyrophosphorylases, directly affects starch synthesis and leads to an accumulation of abundant sugars in corn [16,17]. Therefore, studies on the regulation of glucose metabolism-related genes provide an important foundation for the improvement of the stalk sugar content.

The stalk sugar content of corn is a complex quantitative trait controlled by hereditable factors despite its complex phenotype [18]. The stalk sugar content is strongly associated with kernel filling rate [19]. In sweet sorghum, a reduction in sugar storage in the grain promotes stalk sugar accumulation and increases stem biomass [20]. Elucidation of the molecular mechanism of stalk sugar accumulation in corn is important for breeding new cultivars with an enhanced stalk sugar content. In previous studies, using 202 recombinant inbred lines, the major quantitative trait locus (QTL) qSSC-2.1 (bnlg1909-umc1635) was identified, which explained 13.8% of the phenotypic variance and exhibited high stability. The average stem sugar content of lines carrying the main-effect QTL was 12.5%, and as much as 15% in several lines [3,21]. In addition, QTLs for sugar content has been mapped in sweet sorghum [22]. Two QTLs for stalk sugar content were identified in sweet sorghum and were useful to improve the sugar content by marker-assisted selection [23]. Using a diverse panel of one hundred and twenty-five sorghum accessions, three significant associations for plant height and stem sugar (Brix) traits were detected through association mapping in sweet sorghum [24]. A QTL for stem sugar content on chromosome 3 was identified and explained 25% of the genetic variance [25].

Linkage analysis is limited by parental differences and population size, and the number of localized QTLs is limited [26]. In recent years, genome-wide association analysis (GWAS) has proven to be an effective method to study complex traits [27]. Important progress has been achieved in corn and a series of candidate genes has been detected. To date, GWAS has been applied to corn in studies of resistance to head smut [28], kernel oil concentration and fatty acid composition [29], drought tolerance [30], cold tolerance [31], and other phenotypic traits [32]. However, GWAS of stalk sugar content-related traits in corn has not been reported previously.

To uncover the molecular regulation mechanism of the stalk sugar content and yield in fresh corn, the identification of the genetic variations and candidate genes responsible for the stalk sugar content and agronomic traits is the key problem. In this study, based on one hundred and eighty-eight sweet, waxy, and hybrid corn accessions differing in genetic background, the stalk sugar content and other agronomic traits (e.g., plant height, whole weight per plant, and ear weight with bract) were investigated in four seasons of two years during the spring and autumn sowing. A GWAS analysis was performed to detect the significant single-nucleotide polymorphisms (SNPs) associated with stalk sugar content and the agronomic traits, and the candidate genes were screened, which provides significant insights into the genomic footprints of the stalk sugar content in fresh corn and facilitates the breeding of corn cultivars with higher stalk sugar content.

## 2. Results

### 2.1. Identification of SNPs

**Figure 1:**
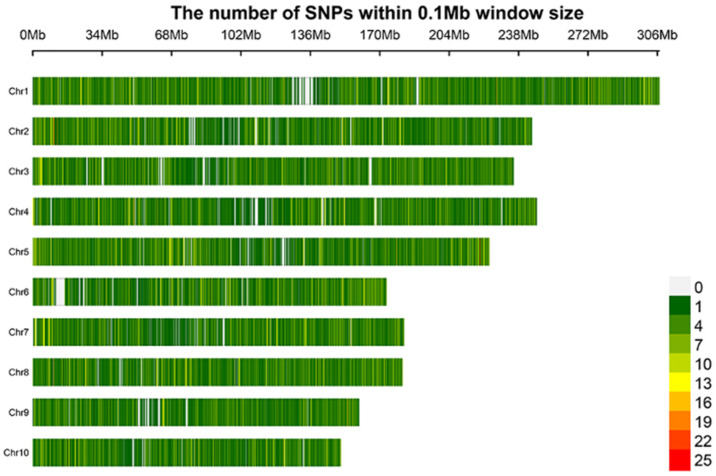
Density of SNPs on the 10 chromosomes of corn within a 0.1 Mb window size. The spectrum column indicates the densities by different colors.

In this study, 188 sweet corn, waxy corn, and hybrid accessions were genotyped. Among them, 41 were sweet corn inbred lines, 74 were waxy corn inbred lines, and 73 were hybrid accessions (Table S1). After comparison with the reference genome (B73 RefGen_v4) and filtering, a total of 36,069 high-quality SNP markers (minimum allele frequency > 0.05 and missing data < 30%) were retained. Among these SNPs, 35,824 markers were distributed on 10 chromosomes (Figure 1). After annotation with ANNOVAR, 5590 (14.99%) SNPs were determined to be located in coding regions, comprising 2133 (5.72%) nonsynonymous SNPs and 3457 (9.27%) synonymous SNPs (Figure S1). In addition, 5698 (15.28%) and 4418 (11.85%) SNPs were located in intronic and intergenic regions, respectively. Examination of the SNP mutation types (Figure S2) revealed that the transition/transversion ratio was 2.81 (Table S2).

### 2.2. Population Structure, Relative Kinship, and LD Decay

**Figure 2:**
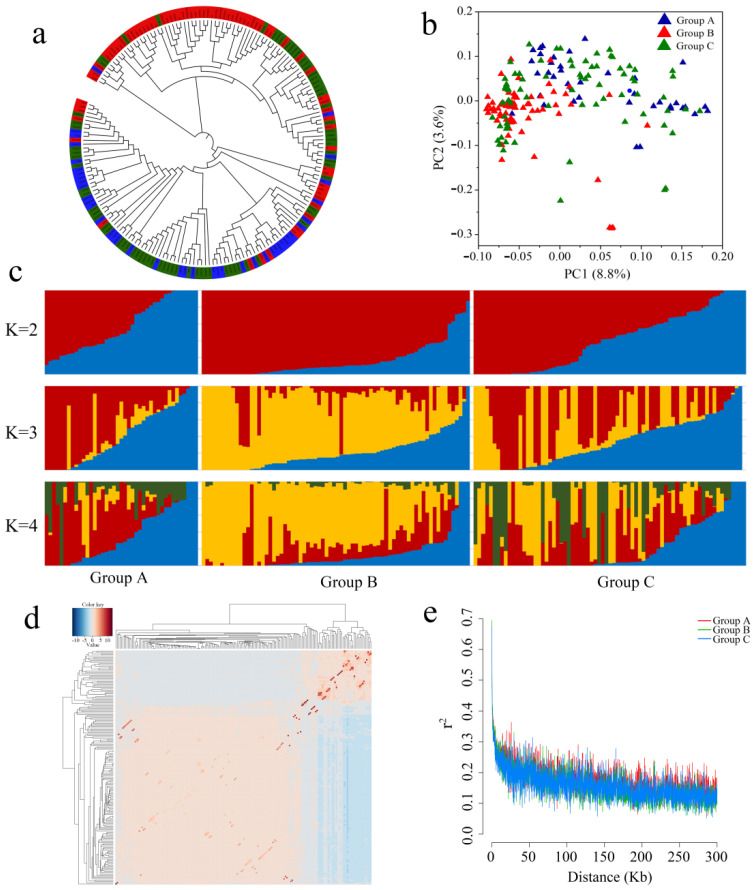
Population structure, principal component analysis, and linkage disequilibrium decay of the 188 fresh corn accessions. (**a**) Phylogenetic tree inferred from the 36,069 high-quality SNPs. Blue, red, and green colors represent sweet corn, waxy corn, and hybrid accessions, respectively. (**b**) Principal component analysis plot of the 188 fresh corn accessions on the first two principal components (PC1 and PC2). Groups A, B, and C include sweet corn, waxy corn, and hybrid accessions, respectively. (**c**) Population structure analysis of the corn accessions performed with ADMIXTURE software. Different results are displayed when *K* = 2, 3, or 4. (**d**) Relative kinship heatmap of the corn accessions. (**e**) Genome-wide linkage disequilibrium (LD) decay (measured by *r*^2^) of the different groups.

To understand the population structure of the 188 fresh corn accessions, we first constructed a phylogenetic tree through the maximum likelihood method using 36,069 high-quality SNPs. The sweet corn and waxy corn accessions were mainly clustered into two subclasses, and most hybrid accessions were classified together with sweet corn accessions (Figure 2a), which indicated that sweet corn constituted a higher proportion of the genetic background of the hybrid accessions. Then, a two-dimensional principal component analysis (PCA) was performed using the GCTA software (Figure 2b and Figure S3). The first and second principal components (PC1 and PC2) explained 8.8% and 3.6% of the total variations, respectively. The PC1 separated the sweet corn (group A) and waxy corn (group B) accessions, and the hybrid accessions (group C) were placed near the sweet corn accessions, which was consistent with the relationships represented in the phylogenetic tree. To further analyze the genetic background of the sweet corn (group A), waxy corn (group B), and hybrid (group C) accessions, a population structure analysis was conducted through ADMIXTURE software (Figure 2c and Figure S4, Table S3), which also supported the relationships suggested from the phylogenetic analysis and PCA. Thus, fresh corn was indicated to be the core breeding material among the study population, and the genetic relationships among the germplasm were complicated likely as a result of long-term crossing and selection. To further clarify the relationships among the accessions used in this study, we evaluated the relative kinship of the 188 corn accessions using the TASSEL software (Figure 2d). Most accessions were weakly related to each other except for a small number of samples, which did not affect the subsequent association analysis. The estimated linkage disequilibrium (LD) decay in the three groups was similar and decayed to *r*^2^ = 0.2 at about 50 kb (Figure 2e).

### 2.3. Selective Sweep Analysis

**Figure 3:**
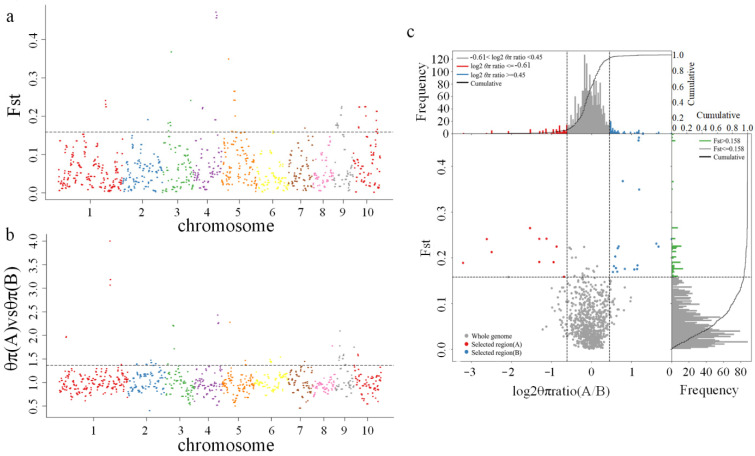
Selective sweeps between sweet corn and waxy corn accessions. (**a**) Distribution of the selective sweeps identified by genetic differentiation coefficient (*F*_ST_). The threshold is represented by a dashed line (top 5% of *F*_ST_ values). (**b**) Distribution of the selective sweeps identified by nucleotide diversity (π_A_/π_B_). The threshold is represented by a dashed line (top 5% of π_A_/π_B_ values). (**c**) Selective sweep regions with the top 5% of *F*_ST_ and π_A_/π_B_ values.

To determine the possible selective signals during the breeding history of the study population, we performed a selective sweep analysis with the genetic differentiation coefficient (*F*_ST_) and nucleotide diversity between the sweet corn (group A) and waxy corn (group B) accessions (π_A_/π_B_). In total, 18 and 20 significant selective regions were detected from the *F*_ST_ and π_A_/π_B_ values, respectively (Figure 3). A total of 85 and 110 genes were located in these selective regions, respectively (Tables S4 and S5). Among the selective regions, six were detected both from the *F*_ST_ and π_A_/π_B_, and 23 genes were located in these regions (Table 1), including a cation transport protein (*Zm00001d040627*), proline-rich receptor-like protein kinase (*Zm00001d033104*), and zinc finger protein (*Zm00001d052883*). A gene ontology (GO) analysis of all selective genes revealed that 53 GO terms were enriched (Figures S5 and S7). A Kyoto Encyclopedia of Genes and Genomes (KEGG) pathway analysis indicated that “ribosome” and “terpenoid backbone biosynthesis” were the top two enriched pathways (Figures S6 and S8). These results indicated that specific genes might have played important roles in the natural and artificial selection of the studied sweet corn and waxy corn accessions.

### 2.4. Phenotyping Results

Stalk sugar content and related agronomic traits from four seasons in two years were investigated (Table S6). The eight traits (summarized in Tables S7–S10) each exhibited broad continuous variation in all environments (Figures S9–S16). Significant differences in sugar content traits were observed among the inbred lines (Table S10), including the sugar content of the stalk (SCS), sugar content in the lower ear parts (SCLEP), three-node sugar content in the ear (TNSCE), and sugar content in the upper ear parts (SCUEP). The mean SCS in the spring and autumn of 2020 was 9.14% and 8.15%, with ranges between 3.6–18.1% and 2.7–17.6%, respectively. The mean SCS in the spring and autumn of 2021 was 8.73% and 7.48%, with ranges between 1.9–16.1% and 2.7–17.6%, respectively (Table S11). Thus, considerable genetic variation in SCS was indicated among the accessions. This genetic variation provided a genetic basis for the improvement of SCS in corn. In addition, all agronomic traits showed continuous and approximately normal distributions (Figures S9–S16) and thus were suitable for GWAS.

Based on the phenotypic data measured in the four environments, the effects of genotype on SCS and the other traits were strongly significant, but also were affected by seeding year and location (Table S12). The broad-sense heritability was greater than 90% for the whole weight per plant (WWP), SCS, SCLEP, TNSCE, and SCUEP (Table S12). These results indicated that the stalk sugar content of fresh corn was mainly controlled by genetic factors and thus was suitable for further association analysis. 

### 2.5. Genome-Wide Association Analysis

**Figure 4:**
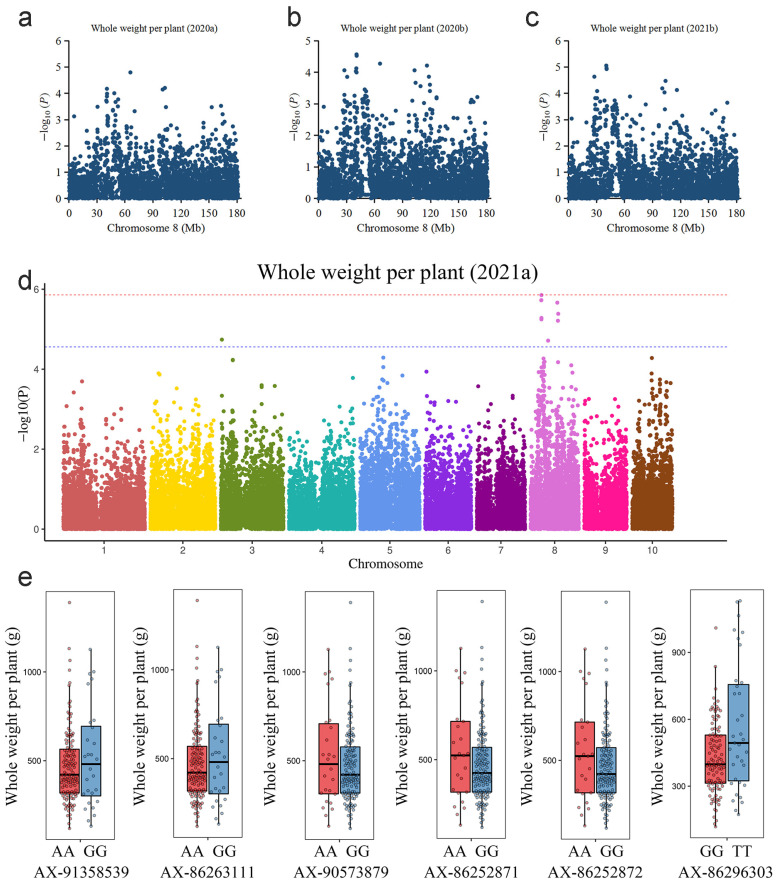
Genome-wide association analysis of whole weight per plant (WWP) using the mixed linear model. The dashed line indicates the significance threshold. (**a**) Local Manhattan plot of WWP from spring 2020 (2020a) on chromosome 8. (**b**) Local Manhattan plot of WWP from autumn 2020 (2020b) on chromosome 8. (**c**) Local Manhattan plot of WWP from autumn 2021 (2021b) on chromosome 8. (**d**) Local Manhattan plot of WWP from spring 2021 (2021a). (**e**) Box plots for WWP based on the different genotypes of important significant SNPs detected in multiple environments.

In this study, the generalized linear model (GLM) and mixed linear model (MLM) models were constructed for the association analysis, but the GLM model showed poor correlations for most traits except the ear node (EN). Therefore, the more reliable MLM model was adopted for most traits and the GLM model was used only for EN (Table S13). Significant signals consistently detected in the different environments were considered to represent high-confidence associations (Table 2). For the agronomic traits, eight significant SNPs associated with plant height (PH) were identified (Figure S17). Among them, three and two significant SNPs were detected in the spring and autumn of 2020, respectively, and the association analysis of the traits from the spring and autumn of 2021 identified two and two significant SNPs, respectively (Table S13). Among these SNPs, one (AX-86251807) was detected in two environments and explained 15.4% and 14.3% of the phenotypic variance. The relevant candidate gene (*Zm00001d020297*) encoded a calcium-dependent protein kinase (CDPK). Fifteen significant SNPs associated with WWP were identified (Figure 4). Among them, five and one significant SNPs were detected in the spring and autumn of 2020, respectively, and the association analysis of the traits from the spring and autumn of 2021 identified ten and six significant SNPs, respectively (Table S13). Importantly, six SNPs were detected in at least two environments. The candidate genes encoded ATP-binding cassette (ABC) transporter, leghemoglobin reductase, and plant homeobox domain (PHD) finger protein, among others. Most importantly, SNP AX-86252871 was identified in three environments and explained 15.0–15.4% of the phenotypic variance. This SNP was located within the exon of the candidate gene *Zm00001d009167*, which encoded a polygalacturonase belonging to the glycosyl hydrolase family. The accessions carrying AX-86252871-AA had higher WWP than the accessions carrying AX-86252871-GG, and the genotypes carrying the other five significant SNPs (AX-91358539, AX-86263111, AX-90573879, AX-86252872, and AX-86296303) differed in WWP (Figure 4E). Twenty-four significant SNPs associated with EWB were detected (Figure S18, Table S13). The relevant candidate genes encoded lipid phosphate phosphatase, premnaspirodiene oxygenase, E3 ubiquitin-protein ligase, acyl transferase, and zinc finger protein, among others. With regard to EN, 11 significant SNPs were detected with the GLM model (Figure S19, Table S13). The relevant candidate genes encoded serine/threonine protein kinase, glycoprotein 3-alpha-L-fucosyltransferase, and a MADS-box transcription factor, among others.

**Figure 5:**
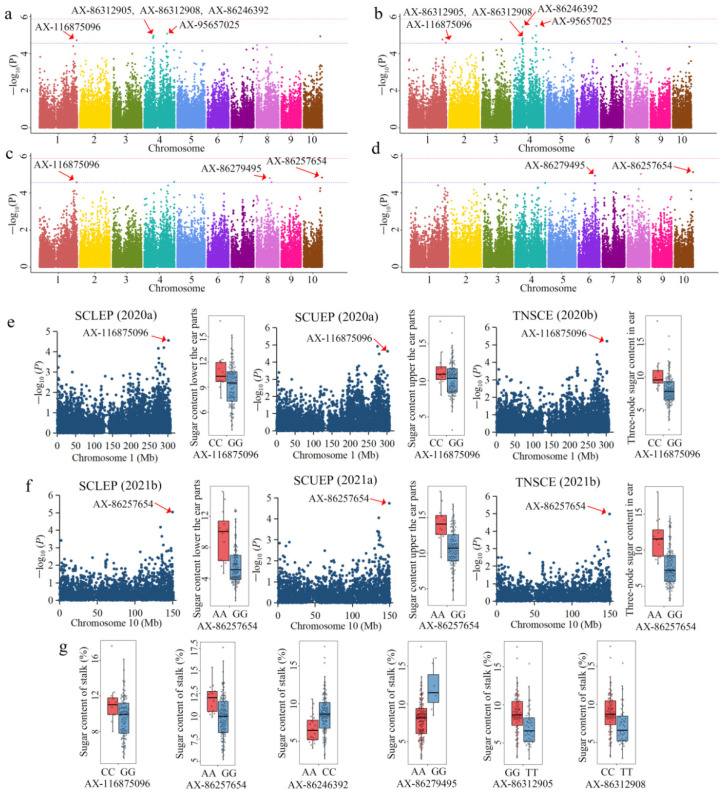
Identification of significant SNPs associated with sugar content of the stalk (SCS) by genome-wide association analysis. (**a**–**d**) Manhattan plots of SCS from spring 2020, autumn 2020, spring 2021, and autumn 2021, respectively. (**e**) Important significant SNP AX-116875096 detected by association analysis of sugar content in the lower ear parts (SCLEP), three-node sugar content in the ear (TNSCE), and sugar content in the upper ear parts (SCUEP). Box plots of the traits are based on the different genotypes of AX-116875096. (**f**) Important significant SNP AX-86257654 detected by association analysis of SCLEP, TNSCE, and SCUEP. Box plots of the traits are based on the different genotypes of AX-86257654. (**g**) Box plots for sugar content of the stalk based on the different genotypes of important significant SNPs detected in multiple environments.

To explore the candidate genes associated with stalk sugar content, we conducted a GWAS on the sugar content of the different stalk parts. For SCS, 18 significant SNPs were identified, which were distributed on chromosomes 1, 3, 4, 6, 7, 8, and 10 (Figure 5). Additionally, six and twelve significant SNPs were detected in the spring and autumn of 2020, respectively, and the association analyses of the SCS from the spring and autumn of 2021 identified five and three significant SNPs, respectively (Table S13). Among these SNPs, seven were detected in multiple environments and eleven were detected in a single environment. The seven significant SNPs explained 13.68% to 17.82% of the phenotypic variation and could be regarded to be stable genetic markers. Most importantly, the significant SNP AX-116875096 on chromosome 1 was detected in three environments in both years and explained 13.68–15.15% of the phenotypic variance. The significant SNP AX-116875096 was located in the 5′ untranslated region (UTR) of the candidate gene *Zm00001d034759*, which encoded an acyl-CoA-binding domain-containing protein. Through phenotypic analysis of the accessions with different genotypes, the accessions carrying AX-116875096-CC were shown to have higher stalk sugar content than the accessions carrying AX-116875096-GG (Figure 5g). In addition, the association analysis of SCLEP, TNSCE, and SCUEP also detected the significant SNP AX-116875096 (Figure 5e, Table S13), which demonstrated that the significant SNP AX-116875096 and candidate gene *Zm00001d034759* were associated with high credibility. An additional important significant SNP, AX-86257654, was also detected in the association analysis of SCLEP, TNSCE, and SCUEP (Figure 5f, Table S13). The sugar content of the accessions carrying AX-86257654-AA was higher than that of the accessions harboring AX-86257654-GG (Figure 5f,g). The relevant candidate gene (*Zm00001d026668*) encoded the receptor protein kinase TMK1. In addition, the sugar content of the accessions with different genotypes of the significant SNPs AX-86246392, AX-86279495, AX-86312905, and AX-86312908 also be analyzed, which shows that the accessions carrying AX-86246392-CC, AX-86279495-GG, AX-86312905-GG, and AX-86312908-CC had higher stalk sugar content (Figure 5g).

In the association analysis for SCLEP, three, twelve, four, and three significant SNPs were detected in the four environments, respectively (Table S13). Among these SNPs, four significant SNPs were detected in two environments. AX-116875096 and AX-95657025 were associated with SCLEP in 2020, whereas AX-86279495 and AX-86257654 were associated with SCLEP in 2021 (Figure S20, Table S13). The top four significant SNPs were also detected in the GWAS of SCS and SCUEP (Table S13). The significant SNP AX-95657025 was located in the exon of the candidate gene *Zm00001d052289*, which encoded pyruvate dehydrogenase, and AX-86279495 was located in the 3′ UTR of the candidate gene *Zm00001d010314*, which encoded an unknown protein. For SCUEP, twenty-five significant SNPs were identified (Figure S21, Table S13), of which eleven SNPs were detected in at least two environments, including AX-86308222 and AX-86319101, which were detected in three environments. AX-86308222 was located upstream of the candidate gene *Zm00001d033915*, which encoded a glycerol-3-phosphate 2-*O*-acyltransferase, and AX-86319101 was located in the intron of the candidate gene *Zm00001d008334*, which encoded a pectinesterase precursor. The accessions carrying AX-86308222-TT and AX-86319101-CC had a higher sugar content in the upper ear parts than the accessions carrying AX-86308222-GG and AX-86319101-TT (Figure S21). The GWAS analysis of TNSCE identified 15 significant SNPs (Table S13). Among these SNPs, eight significant SNPs were detected in two environments, of which five SNPs were also identified in the GWAS of SCS, and AX-86263525, AX-91346570, and AX-86258646 were the other three significant SNPs (Figure S22, Table S13). The SNP AX-86263525 was located in the exon of the candidate gene *Zm00001d042287*, which encoded an MYB transcription factor, and AX-86258646 was located in the exon of the candidate gene *Zm00001d025992*, which encoded an unknown protein. Analysis of the sugar content of the accessions with different genotypes revealed that the accessions carrying AX-86263525-TT and AX-86258646-CC had a higher sugar content in the upper ear parts than the accessions carrying AX-86263525-CC and AX-86258646-TT (Figure S22). In this study, the Q-Q plots were shown in the Figures S17–S20 and S23–S26.

### 2.6. qRT-PCR Analysis of Candidate Genes

**Figure 6:**
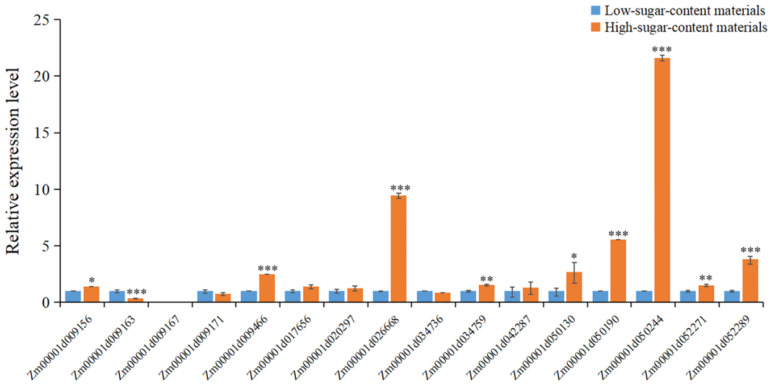
The expression levels of important candidate genes in two fresh corn materials with significantly different stalk sugar content by quantitative RT-PCR. Error bars were used to express the standard deviation of three biological replications. * Represents *p* < 0.05, ** represents *p* < 0.01, *** represents *p* < 0.001.

To better understand the function of candidate genes and genetic variants, the expression levels of sixteen important candidate genes were verified by a quantitative real-time PCR (qRT-PCR) in two fresh corn materials with significantly different stalk sugar content. The average stalk sugar content in multiple environments of the high-sugar content material was 15.5%, while the average stalk sugar content of the low-sugar content material was 3.7%. Based on the qRT-PCR results, we found that the expression levels of *Zm00001d009466*, *Zm00001d026668*, *Zm00001d034759*, *Zm00001d050190*, *Zm00001d050244*, *Zm00001d052271*, *Zm00001d052289* in the high-sugar content material were significantly higher than the low-sugar content material, especially *Zm00001d026668, Zm00001d050190* and *Zm00001d050244* (Figure 6). Furthermore, the significant SNP AX-86257654 was associated with *Zm00001d026668*. We found that the high-sugar content material carried AX-86257654-AA and the low-sugar content material carried AX-86257654-GG. Additionally, the expression levels of *Zm00001d050244* showed an obvious change between the high-sugar content material and the low-sugar content material. The high-sugar content material carried AX-86246392-CC and the low-sugar content material carried AX-86246392-AA. Moreover, the expression level of *Zm00001d050190* in the high-sugar content material carrying AX-86312905-GG was higher than the low-sugar content material carrying AX-86312905-TT. Additionally, *Zm00001d009163* had a significantly higher expression level in low-sugar content materials, and *Zm00001d009167* did not express in the two materials (Figure 6). 

## 3. Discussion

Sugars are the primary products of photosynthesis and accumulate in sinks, including the stalk. Studying the sugar content and yield-related traits in corn is helpful to improve the yield and quality of fresh corn, which is beneficial to the breeding of crosses with higher stalk sugar content and yield. The stalk sugar content also directly affects the application value of fresh corn as a biofuel feedstock. Research on the stalk sugar content of fresh corn may improve the efficiency of corn utilization and expand its application for green silage and biofuel production. The stalk sugar content may also influence the sustainability features of the processes of green silage and biofuels, using advanced sustainability assessment tools is beneficial to evaluate the application value of fresh corn as biofuel production [38]. In recent years, GWAS has become an important method for the discovery of crucial candidate genes associated with complex traits [39]. Previously, QTL mapping was used to locate genes associated with stalk sugar content [3,21]. In the present study, to detect candidate genes associated with stalk sugar content and related agronomic traits in fresh corn, we performed GWAS based on GLM and MLM models. The GLM model led to many false positives, whereas the MLM model effectively reduced the number of false positives, as reported in previous studies [40,41]. Numerous significant SNPs were detected in more than one environment, and thus these genetic variations and candidate genes are of substantial research and practical value.

The QTLs that contribute to the stalk sugar content also affect the leaf area of corn [21]. In addition, yield is critical for the use of fresh corn in silage. In the association analysis of PH and WWP, reliable significant SNPs were simultaneously detected in multiple environments. Calcium-dependent protein kinase (CDPK) plays an important role in plant growth, development, and stress response [42]. Previous studies in sugarcane have shown that CDPK may phosphorylate and regulate sucrose synthase and sucrose phosphate synthase, and therefore is important for sucrose accumulation [9,10]. In the current study, the candidate gene *Zm00001d020297* encodes a CDPK, which was identified in two environments and might be a crucial candidate gene regulating plant height. Moreover, the alleles of AX-86252871 had differential effects on WWP, the accessions carrying the allele AX-86252871-AA had a higher WWP (Figure 4e). AX-86252871 is located in the exon region of candidate gene *Zm00001d009167*, which encoded a polygalacturonase and belonged to the glycosyl hydrolase family. Polygalacturonases are ubiquitous in higher plant cells and are strongly associated with plant growth and development owing to their functions in cell wall degradation, promotion of cell division, and fruit ripening [43]. Ethylene can promote the expression of polygalacturonase (PG) and plays a crucial role in sucrose metabolism in blueberries [44]. A previous study reported that the PHD finger protein MePHD1 negatively regulates the transcript level of the *MeAGPS1a* gene, which encods an ADP-glucose pyrophosphorylase (AGPase) [45]. Our study found that the accessions carrying AX-86296303-TT had a higher WWP than the accessions carrying AX-86296303-GG (Figure 4e), which was significantly associated with candidate gene *Zm00001d009466* encoding a PHD finger protein. Additionally, we also identified a candidate gene (*Zm00001d009156*) encoding an ATP-binding cassette (ABC) transporter through significant SNP AX-91358539. The study in *Arabidopsis* has shown that ABC transporters are involved in the transport of lipids, glycosides, hormones, and so on [46]. The results showed that the whole weight per plant (WWP) of the accessions carrying AX-91358539-GG was higher than the accessions carrying AX-91358539-AA (Figure 4e), which might directly influence the function of the ABC transporter and ultimately affect the whole weight per plant.

Sugar is an important photosynthate and is the main product of photosynthesis transported in the plant body [47]. In this study, a suite of significant SNPs and candidate genes associated with SCS (sugar content of the stalk) was detected with high confidence. Among these SNPs, the significant SNP AX-95657025 was located in the first exon of the candidate gene *Zm00001d052289*, which encodes a pyruvate dehydrogenase involved in energy production and conversion. Pyruvate dehydrogenase is a crucial enzyme required for glucose metabolism, which converts pyruvate to acetyl CoA to complete the tricarboxylic acid cycle [15]. Mutation of genes encoding components of the pyruvate dehydrogenase complex affects enzymatic activity and plant growth [48]. We found that the expression level of *Zm00001d052289* in the high-sugar content material was significantly higher than in the low-sugar content material (Figure 6), and future research should be focused on whether the variation of the SNP AX-95657025 affects the function of the candidate gene *Zm00001d052289*. Additionally, previous studies have reported that Acyl-CoA-binding domain-containing protein (ACBD) is required for the transferal of glucosylceramide [49], and plays an important role in plant growth, development, and stress response [50,51]. In the current study, four traits related to the stalk sugar content (SCS, SCLEP, TNSCE, and SCUEP) were associated with the significant SNP AX-116875096, which was also detected in multiple environments. The SNP AX-116875096 was located in the 5′ UTR of *Zm00001d034759*, which encodes an ACBD protein. The accessions carrying AX-116875096-CC had higher sugar content in the stalk (Figure 5e), and the expression level of *Zm00001d034759* was higher in the high-sugar content material (Figure 6), which reflected that the genetic variation might influence the expression of the candidate gene *Zm00001d034759*.

Analysis of the expression profile of signal transduction components in sugarcane has shown that the receptor protein kinase may regulate sucrose metabolism [11]. In the present study, the candidate gene *Zm00001d026668* encoding a receptor protein kinase TMK was identified through the significant SNP AX-86257654. AX-86257654 was detected in four traits (SCS, SCLEP, TNSCE, and SCUEP) and multiple environments, which had higher credibility. We found that the accessions carrying AX-86257654-AA had higher talk sugar content than the accessions carrying AX-86257654-GG (Figure 5g). The expression level of *Zm00001d026668* in the high-sugar content material was significantly higher than the low-sugar content material (Figure 6), while the high-sugar content material carrying AX-86257654-AA and the low-sugar content material carrying AX-86257654-GG. Moreover, a candidate gene (*Zm00001d042287*) encoding an MYB transcription factor was identified in multiple environments, and the SNP AX-86263525 was located in the exon of *Zm00001d042287*. The alleles of AX-86263525 had different effects on stalk sugar content and explained 15.16%–16.57% of the phenotypic variance. The accessions carrying AX-86263525-TT had higher stalk sugar content than the accessions carrying AX-86263525-CC (Figure S22), and the expression level of *Zm00001d042287* was significantly higher in the high-sugar content material, reflecting that the mutation of AX-86263525 might affect the function of *Zm00001d042287*. The studies have reported that MYB transcription factors are involved in the biosynthesis of the water-soluble polysaccharide and MYB46 directly regulates the three *CESA* genes, which are essential for cellulose production [12,13]. We also found that *Zm00001d050244* had a significantly higher expression level in the high-sugar content material (Figure 6), which encoded a DEAD-box ATP-dependent RNA helicase, while the high-sugar content material carried AX-86246392-CC and the high-sugar content material carried AX-86246392-AA (Figure 5g). The DEAD-box ATP-dependent RNA helicase plays an important role in regulating translation initiation and growth in plants [52]. Thus, we inferred that *Zm00001d050244* might take part in sugar metabolism by regulating the translation of other genes. In addition, candidate genes encoding the pectinesterase precursor and cell wall protein were also detected in this study.

Differing from the QTL mapping in previous studies, our study detected the candidate gene responsible for the stalk sugar content of fresh corn through the genome-wide association analysis. Despite many significant genetic variations and candidate genes detected in the present study, which have a potential value for the future breeding of fresh corn, there is still room to further validate the true phenotypic effects of the corresponding two haplotypes through transgenic experiments. Hence, further investigation of the molecular mechanism of the genetic variations and candidate genes associated with the stalk sugar content in fresh corn is required.

## 4. Materials and Methods

### 4.1. Plant Materials

The plant seed materials included 188 sweet corn, waxy corn, and hybrid accessions. Among them, 41 were sweet corn inbred lines, 74 were waxy corn inbred lines, and 73 were hybrid accessions. Seeds were obtained from southeastern China, northern China, and the Huang-Huai-Hai region of eastern China.

### 4.2. Trait Measurements and Analysis

Seeds of the experimental materials were sown in an experimental field at Dongyang, Zhejiang, China (29°16′ N, 120°19′ E) in the spring and autumn of 2020 and 2021. A completely randomized design with three replications was used in this study. Each replicate comprised two rows of each accession of 3 m in length, and the plant density was 0.75 m × 0.3 m. Field management (e.g., watering, fertilization, and weed management) was applied uniformly throughout the growing period.

Before tasseling, 10 plants of each accession exhibiting normal growth and development were selected for measurement of stalk sugar content and agronomic traits. Artificial pollination was performed to prevent pollen mixing. In the harvest season for fresh corn, eight plants per growth period were selected for measurement of plant height (PH), whole weight per plant (WWP), ear weight with bract (EWB), ear node (EN), sugar content of the stalk (SCS), sugar content in the lower ear parts (SCLEP), three-node sugar content in the ear (TNSCE), and sugar content in the upper ear parts (SCUEP). For estimation of the sugar content, we extracted the whole stem juice from each plant, and after thorough mixing, the sugar content was determined with a hand-held sugar meter (PAL-1, Atago, Tokyo, Japan). To minimize measurement error, the stem juice of each plant was measured three times, and the average was taken as the sugar content of each individual. Frequency distribution analysis, correlation analysis, and variance analysis of the data were performed with SPSS software (version 22.0).

### 4.3. Genotypic Data

The 188 sweet corn, waxy corn, and hybrid accessions were genotyped with the Axiom^®^ Maize56K SNP Array, which includes 56,000 SNPs in total. After comparison with the corn genome (B73 RefGen_v4), SNP markers with minimum allele frequency >0.05 and missing data <30% were filtered using TASSEL software [53]. Annotation of the SNPs was performed with ANNOVAR [54]. A total of 36,069 SNPs were used for subsequent analysis.

### 4.4. Population Structure Analysis, Linkage Disequilibrium, and Relative Kinship Estimation

A phylogenetic tree was constructed from a data set of 36,069 SNPs using the maximum likelihood method in FastTree 2.1.9 [55]. The tree was colored using iTQL (https://itol.embl.de/ (accessed on 27 October 2022)). Principal component analysis (PCA) was conducted using GCTA software (version 1.92.0) [56]. The first two eigenvectors were selected to generate a scatterplot of the principal component scores to visualize similarities among the accessions in two dimensions. The ADMIXTURE software was used to investigate the population structure [57]. The relative kinship matrix (K) was calculated using TASSEL software [53]. Linkage disequilibrium (LD) was calculated using PopLDdecay software [58]. 

### 4.5. Detection of Selective Sweeps 

The selective sweeps between sweet corn (group A) and waxy corn (group B) were detected using VCFtools [59]. First, the nucleotide diversity of the sweet corn and waxy corn accessions (π_A_ and π_B_, respectively) was calculated using a 100 kb window and a step size of 10 kb. Then, π_A_/π_B_ was calculated and the windows, with the top 5% largest π_A_/π_B_ values were identified as selective sweeps. In addition, the genetic differentiation (*F*_ST_) among sweet corn (group A) and waxy corn (group B) was determined using a 100 kb window and a step size of 10 kb, and the windows with the top 5% largest *F*_ST_ values were identified as selective sweeps. To explore the function of selected genes located in the selective regions, the AgriGO analysis toolkit [60] and the Kyoto Encyclopedia of Genes and Genomes (KEGG) database were used to perform gene ontology (GO) and KEGG pathway analyses.

### 4.6. Genome-Wide Association Analysis

Plant height (PH), whole weight per plant (WWP), ear weight with bract (EWB), ear node (EN), sugar content of the stalk (SCS), sugar content in the lower ear parts (SCLEP), three-node sugar content in the ear (TNSCE), and sugar content in the upper ear parts (SCUEP) recorded in four environments were used for GWAS analysis. For the association analysis, a generalized linear model (GLM) and mixed linear model (MLM) were generated with TASSEL [53]. Manhattan plots and quantile-quantile plots were generated with R software based on the *p*-values from the association analysis [61]. The significant association threshold was defined as 0.05/*n* and 1/*n*, where *n* is the number of SNPs [62].

### 4.7. RNA Extraction and Quantitative RT-PCR Analysis

To verify the expression levels of important candidate genes, total RNA was extracted from two fresh corn materials with significantly different stalk sugar content using the RNAprep Pure Plant Kit (TIANGEN, Beijing, China): the high-sugar content material LQ5 from a domestic hybrid second cycle line and the high-sugar content material SH23 from a domestic inbred line. A total of 1 ug RNA was used to synthesize cDNA according to the instructions of a PrimeScript RT Reagent Kit (TaKaRa, China). The qRT-PCR reactions were performed through the SYBR-Green PrimeScript RT-PCR Kit (Takara) following the manufacturer’s instructions. *GAPDH* was used as the reference gene for the qRT-PCR. The original data were processed by the 2^−ΔΔCt^ method, where Ct is the threshold cycle [63], and the primers for candidate genes were shown in Table S14.

## 5. Conclusions

In this study, genome-wide association analysis was used to detect significant SNPs and candidate genes associated with stalk sugar content and agronomic traits. A total of 92 significant associated SNPs were identified. Among them, 24 significant SNPs and 22 candidate genes were identified in multiple environments, including CDPK, MYB transcription factor, pyruvate dehydrogenase, and so on. Most importantly, accessions differing in genotype for several significant SNPs showed significant phenotypic differences, indicating that the genetic variations in candidate genes might be closely related to the phenotypic traits. The expression levels of six important candidate genes were significantly different between the high-sugar content material and the low-sugar content material. In general, the significant genetic variations might be useful in molecular marker-assisted breeding, and the investigation of candidate genes may provide important insights into the mechanism of stalk sugar content in fresh corn, which establishes the foundation for fresh corn breeding. Future research should focus on illustrating how the genetic variations and candidate genes regulate the stalk sugar content in fresh corn, and concentrate on breeding new varieties with higher stalk sugar content.

## Figures and Tables

**Table 1:** Summary of the genes located in the selective sweep regions both with the top 5% of *F*_ST_ and π_A_/π_B_ between sweet and waxy corn accessions.

Gene ID	Chromosome	Start	End	Gene Function
*Zm00001d033104*	1	250291343	250304767	Proline-rich receptor-like protein kinase PERK
*Zm00001d033105*	1	250320688	250322547	Ribosomal protein
*Zm00001d033106*	1	250323656	250324358	Unknown
*Zm00001d033107*	1	250328690	250338437	Sulfated surface glycoprotein
*Zm00001d033108*	1	250339356	250347246	Lysine--tRNA ligase
*Zm00001d033109*	1	250346963	250360412	P-loop containing nucleoside triphosphate hydrolase superfamily protein
*Zm00001d040502*	3	47172433	47173432	Early nodulin-like protein
*Zm00001d040503*	3	47223813	47241740	Retrovirus-related Pol polyprotein
*Zm00001d040504*	3	47292181	47293995	Lamin-like protein
*Zm00001d040627*	3	54948300	54950704	Cation transport protein
*Zm00001d040628*	3	55023821	55026070	Unknown
*Zm00001d052883*	4	203324761	203327583	Zinc finger protein
*Zm00001d052885*	4	203405706	203407600	40S ribosomal protein
*Zm00001d052886*	4	203407872	203412281	Sulfotransferase
*Zm00001d052888*	4	203494690	203511433	Polyadenylate-binding protein
*Zm00001d045596*	9	28028596	28031674	BAG family molecular chaperone regulator
*Zm00001d045597*	9	28031820	28047755	Rho GTPase-activating protein
*Zm00001d045598*	9	28060153	28063330	Unknown
*Zm00001d045599*	9	28082328	28086696	Monocopper oxidase-like protein
*Zm00001d045600*	9	28126449	28127152	NAC domain-containing protein
*Zm00001d046109*	9	63900291	63900708	60S ribosomal protein
*Zm00001d046111*	9	63970902	63971348	Unknown
*Zm00001d046112*	9	63973033	63977492	Spermidine synthase

**Table 2:** Significant SNPs and candidate genes simultaneously identified in different environments.

Trait	Chromosome	SNP	Position	−log10 (P)	Marker *R*^2^	Location	Gene ID	Gene Function	Locus Tag	Literature
PH	7	AX-86251807	104761239	4.71	15.4%	intronic	*Zm00001d020297*	Calcium-dependent protein kinase	LOC100194392	None
WWP	8	AX-86296303	65780194	4.80	15.3%	intergenic	*Zm00001d009466*	PHD finger protein	None	None
WWP	8	AX-91358539	39945148	5.72	17.2%	intronic	*Zm00001d009156*	Protein ABC transporter	LOC103635101	None
WWP	8	AX-86263111	40371639	5.73	16.9%	exonic	*Zm00001d009163*	Leghemoglobin reductase	LOC100501719	None
WWP	8	AX-90573879	40619812	5.85	17.5%	intergenic	*Zm00001d009165*	Unknown	None	None
WWP	8	AX-86252871	40632157	4.96	15.0%	exonic	*Zm00001d009167*	Polygalacturonase	LOC103635108	None
WWP	8	AX-86252872	40752630	4.92	15.0%	exonic	*Zm00001d009171*	Mitochondrial carrier protein	LOC100274176	None
SCS	1	AX-116875096	301500710	4.72	15.2%	5′UTR	*Zm00001d034759*	Acyl-CoA-binding domain-containing protein	LOC100384139	[33]
SCS	4	AX-86312905	71985807	4.90	15.7%	intergenic	*Zm00001d050190*	Vegetative cell wall protein	LOC100191378	None
SCS	4	AX-86312908	71985862	4.84	15.6%	intergenic	*Zm00001d050190*	Vegetative cell wall protein	LOC100191378	None
SCS	4	AX-86246392	75672064	5.44	17.8%	3′UTR	*Zm00001d050244*	DEAD-box ATP-dependent RNA helicase	LOC100279228	None
SCS	4	AX-95657025	186090916	5.49	15.3%	exonic	*Zm00001d052289*	Pyruvate dehydrogenase 1	LOC732791	[34]
SCS	8	AX-86279495	109191809	5.02	15.0%	3′UTR	*Zm00001d010314*	Unknown	LOC100272847	None
SCS	10	AX-86257654	149652762	5.12	15.9%	3′UTR	*Zm00001d026668*	Receptor protein kinase TMK1	LOC100192365	None
SCLEP	1	AX-116875096	301500710	4.65	15.2%	5′UTR	*Zm00001d034759*	Acyl-CoA-binding domain-containing protein	LOC100384139	[33]
SCLEP	4	AX-95657025	186090916	4.87	13.4%	exonic	*Zm00001d052289*	Pyruvate dehydrogenase 1	LOC732791	[34]
SCLEP	8	AX-86279495	109191809	5.63	17.1%	3′UTR	*Zm00001d010314*	Unknown	LOC100272847	None
SCLEP	10	AX-86257654	149652762	5.04	15.7%	3′UTR	*Zm00001d026668*	Receptor protein kinase TMK1	LOC100192365	None
SCUEP	4	AX-86246392	75672064	5.03	16.3%	3′UTR	*Zm00001d050244*	DEAD-box ATP-dependent RNA helicase	LOC100279228	None
SCUEP	8	AX-86319101	5579363	5.08	16.9%	intronic	*Zm00001d008334*	Pectinesterase precursor	LOC100191701	[35]
SCUEP	4	AX-86300656	181467257	5.69	18.6%	exonic	*Zm00001d052157*	Unknown	LOC100280272	None
SCUEP	4	AX-86283479	181472159	4.95	16.0%	3′UTR	*Zm00001d052157*	Unknown	LOC100280272	None
SCUEP	4	AX-95657025	186090916	5.27	14.6%	exonic	*Zm00001d052289*	Pyruvate dehydrogenase 1	LOC732791	[34]
SCUEP	5	AX-86293407	203302677	5.42	17.9%	downstream	*Zm00001d017656*	Pigment biosynthesis protein	LOC100282483	None
SCUEP	8	AX-86279495	109191809	5.09	16.7%	3′UTR	*Zm00001d010314*	Unknown	LOC100272847	None
SCUEP	1	AX-86328029	300979520	4.63	14.3%	3′UTR	*Zm00001d034736*	Cysteine--tRNA ligase CPS1	LOC103644288	None
SCUEP	10	AX-86257654	149652762	4.75	15.3%	3′UTR	*Zm00001d026668*	Receptor protein kinase TMK1	LOC100192365	None
SCUEP	1	AX-86308222	278512731	5.22	15.8%	upstream	*Zm00001d033915*	Glycerol-3-phosphate 2-*O*-acyltransferase	LOC103643973	[36]
SCUEP	4	AX-86313840	185513108	5.54	16.6%	3′UTR	*Zm00001d052271*	Protease Do-like 9	LOC103655753	None
TNSCE	4	AX-91346570	68058771	5.00	16.9%	downstream	*Zm00001d050130*	CBS domain-containing protein CBSX1	LOC100282782	None
TNSCE	4	AX-86312905	71985807	5.20	16.9%	intergenic	*Zm00001d050190*	Vegetative cell wall protein	LOC100191378	None
TNSCE	4	AX-86312908	71985862	5.11	16.7%	intergenic	*Zm00001d050190*	Vegetative cell wall protein	LOC100191378	None
TNSCE	4	AX-86246392	75672064	5.22	17.0%	3′UTR	*Zm00001d050244*	DEAD-box ATP-dependent RNA helicase	LOC100279228	None
TNSCE	1	AX-116875096	301500710	5.21	17.1%	5′UTR	*Zm00001d034759*	Acyl-CoA-binding domain-containing protein	LOC100279228	[33]
TNSCE	3	AX-86263525	159083718	5.02	16.6%	exonic	*Zm00001d042287*	MYB transcription factor	LOC541962	None
TNSCE	4	AX-95657025	186090916	5.20	14.4%	exonic	*Zm00001d052289*	Pyruvate dehydrogenase 1	LOC732791	[34]
TNSCE	10	AX-86258646	135575124	5.02	16.4%	exonic	*Zm00001d025992*	Unknown	LOC100276746	[37]

## Data Availability

The original data presented in this study can be accessed at https://ngdc.cncb.ac.cn/gsub/submit/bioproject/list (project ID: PRJCA009494).

## Supplementary Materials

The following are available online at https://www.mdpi.com/article/10.3390/ijms232113490/s1.

## Abbreviations

GWAS	Genome-wide association analysis
SNPs	Single nucleotide polymorphisms
QTL	Quantitative trait locus
MYB	V-myb avian myeloblastosis viral oncogene homolog
LD	Linkage disequilibrium
PCA	Principal components analysis
*F*_ST_	Genetic differentiation coefficient
π_A_/π_B_	Nucleotide diversity between sweet corns (group A) and waxy corns (group B)
KEGG	Kyoto encyclopedia of genes and genomes
GO	Gene ontology
SCS	Sugar content of the stalk
SCLEP	Sugar content in the lower ear parts
TNSCE	Three-node sugar content in the ear
SCUEP	Sugar content in the upper ear parts
WWP	Whole weight per plant
EN	Ear node
PH	Plant height
EWB	Ear weight with bract
GLM	Generalized linear model
MLM	Mixed linear model
UTR	Untranslated region
qRT-PCR	Quantitative real-time PCR
CDPK	Calcium-dependent protein kinase
ABC	ATP-binding cassette
PHD	Plant homeobox domain
ACBD	Acyl-CoA-binding domain-containing protein
CESA	Cellulose synthase

## References

[1] Shi Z.S., Zhong X.M. (2016). Breeding principle and technical skill of fresh corn varieties. J. Maize Sci..

[2] Bian Y.-L., Du K., Wang Y.-J., Deng D.-X. (2009). Distribution of Sugar Content in Corn Stalk. Acta Agron. Sin..

[3] Bian Y., Gu X., Sun D., Wang Y., Yin Z., Deng D., Wang Y., Li G. (2015). Mapping dynamic QTL of stalk sugar content at different growth stages in maize. Euphytica.

[4] Kleinschmit D.H., Schmidt R.J., Jr L.K. (2005). The effects of various antifungal additives on the fermentation and aerobic stability of corn silage. J. Dairy Sci..

[5] Zhang D.Y., Li Z.Q., Liu C.L. (2007). Progress in the study of infection factors on silage quality. Acta Ecol. Anim. Domastici.

[6] Froetschel M., Ely L., Amos H. (1991). Effects of Additives and Growth Environment on Preservation and Digestibility of Wheat Silage Fed to Holstein Heifers. J. Dairy Sci..

[7] Bai Q.L., Chen S.J., Dai J.R. (2007). Stalk quality traits and their correlations of maize inbred lines in China. Acta Agron. Sin..

[8] Esfandabadi Z.S., Ranjbari M., Scagnelli S.D. (2022). The imbalance of food and biofuel markets amid Ukraine-Russia crisis: A systems thinking perspective. Biofuel Res. J..

[9] Hardin S.C., Winter H., Huber S.C. (2004). Phosphorylation of the Amino Terminus of Maize Sucrose Synthase in Relation to Membrane Association and Enzyme Activity. Plant Physiol..

[10] Papini-Terzi F.S., Rocha F.R., Vêncio R.Z., Felix J.M., Branco D.S., Waclawovsky A.J., Del Bem L.E., Lembke C.G., Costa M.D., Nishiyama M.Y. (2009). Sugarcane genes associated with sucrose content. BMC Genom..

[11] Felix J.D.M., Papini-Terzi F.S., Rocha F.R., Vêncio R.Z.N., Vicentini R., Nishiyama M.Y., Ulian E.C., Souza G.M., Menossi M. (2009). Expression Profile of Signal Transduction Components in a Sugarcane Population Segregating for Sugar Content. Trop. Plant Biol..

[12] Kim W.-C., Ko J.-H., Kim J.-Y., Kim J., Bae H.-J., Han K.-H. (2012). MYB46 directly regulates the gene expression of secondary wall-associated cellulose synthases in Arabidopsis. Plant J..

[13] He C., Da Silva J.A.T., Wang H., Si C., Zhang M., Zhang X., Li M., Tan J., Duan J. (2019). Mining MYB transcription factors from the genomes of orchids (Phalaenopsis and Dendrobium) and characterization of an orchid R2R3-MYB gene involved in water-soluble polysaccharide biosynthesis. Sci. Rep..

[14] Yu H., Du X., Zhang F., Zhang F., Hu Y., Liu S., Jiang X., Wang G., Liu D. (2012). A mutation in the E2 subunit of the mitochondrial pyruvate dehydrogenase complex in Arabidopsis reduces plant organ size and enhances the accumulation of amino acids and intermediate products of the TCA Cycle. Planta.

[15] Ohbayashi I., Huang S., Fukaki H., Song X., Sun S., Morita M., Tasaka M., Millar A.H., Furutani M. (2019). Mitochondrial Pyruvate Dehydrogenase Contributes to Auxin-Regulated Organ Development. Plant Physiol..

[16] Kramer V., Shaw J.R., Senior M.L., Hannah L.C. (2014). The sh2-R allele of the maize shrunken-2 locus was caused by a complex chromosomal rearrangement. Theor. Appl. Genet..

[17] Chhabra R., Hossain F., Muthusamy V., Baveja A., Mehta B., Zunjare R.U. (2019). Mapping and validation of Anthocyanin1 pigmentation gene for its effectiveness in early selection of shrunken2 gene governing kernel sweetness in maize. J. Cereal Sci..

[18] Reen R.V., Singleton W.R. (1952). Sucrose content in the stalks of maize inbreds. Agron. J..

[19] Hua H.L., Zhao Q., Jie W., Liu Q.G., Bian Y.L. (2019). Kernel filling characteristics at different grain positions on an ear and their relationship with stalk sugar content in maize. Int. J. Agric. Biol..

[20] Jebril J., Wang D., Rozeboom K., Tesso T. (2021). Grain sink removal increases stalk juice yield, sugar accumulation, and biomass in sweet sorghum [*Sorghum bicolor* (L.) Moench]. Ind. Crops Prod..

[21] Bian Y., Sun D., Gu X., Wang Y., Yin Z., Deng D., Wang Y., Wu F., Li G. (2014). Identification of QTL for stalk sugar-related traits in a population of recombinant inbred lines of maize. Euphytica.

[22] Shiringani A.L., Frisch M., Friedt W. (2010). Genetic mapping of QTLs for sugar-related traits in a RIL population of Sorghum bicolor L. Moench. Theor. Appl. Genet..

[23] Bian Y.L., Seiji Y., Maiko I., Cai H.W. (2006). QTLs for sugar content of stalk in sweet sorghum (*Sorghum bicolor* L. Moench). Agric. Sci. China.

[24] Murray S.C., Rooney W.L., Hamblin M.T., Mitchell S.E., Kresovich S. (2009). Sweet Sorghum Genetic Diversity and Association Mapping for Brix and Height. Plant Genome.

[25] Murray S.C., Sharma A., Rooney W.L., Klein P.E., Mullet J.E., Mitchell S.E., Kresovich S. (2008). Genetic Improvement of Sorghum as a Biofuel Feedstock: I. QTL for Stem Sugar and Grain Nonstructural Carbohydrates. Crop Sci..

[26] Zhang G., Gao M., Zhang G., Sun J., Jin X., Wang C., Zhao Y., Li S. (2013). Association Analysis of Yield Traits with Molecular Markers in Huang-Huai River Valley Winter Wheat Region, China. Acta Agron. Sin..

[27] Flint-Garcia S.A. (2013). Genetics and Consequences of Crop Domestication. J. Agric. Food Chem..

[28] Wang M., Yan J., Zhao J., Song W., Zhang X., Xiao Y., Zheng Y. (2012). Genome-wide association study (GWAS) of resistance to head smut in maize. Plant Sci..

[29] Li H., Peng Z., Yang X., Wang W., Fu J., Wang J., Han Y., Chai Y., Guo T., Yang N. (2012). Genome-wide association study dissects the genetic architecture of oil biosynthesis in maize kernels. Nat. Genet..

[30] Wang X., Wang H., Liu S., Ferjani A., Li J., Yan J., Yang X., Qin F. (2016). Genetic variation in ZmVPP1 contributes to drought tolerance in maize seedlings. Nat. Genet..

[31] Hu G., Li Z., Lu Y., Li C., Gong S., Yan S., Li G., Wang M., Ren H., Guan H. (2017). Genome-wide association study Identified multiple Genetic Loci on Chilling Resistance During Germination in Maize. Sci. Rep..

[32] Tian F., Bradbury P.J., Brown P.J., Hung H., Sun Q., Flint-Garcia S., Rocheford T.R., McMullen M.D., Holland J., Buckler E. (2011). Genome-wide association study of leaf architecture in the maize nested association mapping population. Nat. Genet..

[33] Zhu J., Li W., Zhou Y., Pei L., Liu J., Xia X., Che R., Li H. (2021). Molecular characterization, expression and functional analysis of acyl-CoA-binding protein gene family in maize (*Zea mays*). BMC Plant Biol..

[34] Thelen J.J., Miernyk J.A., Randall D.D. (1999). Molecular cloning and expression analysis of the mitochondrial pyruvate dehydro-genase from maize. Plant Physiol..

[35] Zhang P., Wang H., Qin X., Chen K., Zhao J., Zhao Y., Yue B. (2019). Genome-wide identification, phylogeny and expression analysis of the PME and PMEI gene families in maize. Sci. Rep..

[36] Zhu T., Wu S., Zhang D., Li Z., Xie K., An X., Ma B., Hou Q., Dong Z., Tian Y. (2019). Genome-wide analysis of maize GPAT gene family and cytological characterization and breeding application of ZmMs33/ZmGPAT6 gene. Theor. Appl. Genet..

[37] Li C., Guan H., Jing X., Li Y., Wang B., Li Y., Liu X., Zhang D., Liu C., Xie X. (2022). Genomic insights into historical improvement of heterotic groups during modern hybrid maize breeding. Nat. Plants.

[38] Aghbashlo M., Hosseinzadeh-Bandbafha H., Shahbeik H., Tabatabaei M. (2022). The role of sustainability assessment tools in realizing bioenergy and bioproduct systems. Biofuel Res. J..

[39] Alqudah A.M., Sallam A., Baenziger P.S., Börner A. (2019). GWAS: Fast-forwarding gene identification and characterization in temperate Cereals: Lessons from Barley–A review. J. Adv. Res..

[40] Yang X., Gao S., Xu S., Zhang Z., Prasanna B.M., Li L., Li J., Yan J. (2010). Characterization of a global germplasm collection and its potential utilization for analysis of complex quantitative traits in maize. Mol. Breed..

[41] Cai D., Xiao Y., Yang W., Ye W., Wang B., Younas M., Wu J., Liu K. (2013). Association mapping of six yield-related traits in rapeseed (*Brassica napus* L.). Theor. Appl. Genet..

[42] Zhao P., Liu Y., Kong W., Ji J., Cai T., Guo Z. (2021). Genome-wide identification and characterization of calcium-dependent protein kinase (CDPK) and CDPK-related kinase (CRK) gene families in Medicago truncatula. Int. J. Mol. Sci..

[43] Wang F., Sun X., Liu B., Kong F., Pan X., Zhang H. (2022). A polygalacturonase gene PG031 regulates seed coat permeability with a pleiotropic effect on seed weight in soybean. Theor. Appl. Genet..

[44] Wang S., Zhou Q., Zhou X., Zhang F., Ji S. (2019). Ethylene plays an important role in the softening and sucrose metabolism of blueberries postharvest. Food Chem..

[45] Ma P., Chen X., Liu C., Xia Z., Song Y., Zeng C., Li Y., Wang W. (2018). MePHD1 as a PHD-Finger Protein Negatively Regulates ADP-Glucose Pyrophosphorylase Small Subunit1a Gene in Cassava. Int. J. Mol. Sci..

[46] Li J., Peng Z., Liu Y., Lang M., Chen Y., Wang H., Li Y., Shi B., Huang W., Han L. (2022). Over-expression of peroxisome-localized GmABCA7 promotes seed germination in Arabidopsis thaliana. Int. J. Mol. Sci..

[47] Shi J.G., Cui H.Y., Zhao B., Dong S.T., Liu P., Zhang J.W. (2013). Effect of light on yield and characteristics of grain-filling of summer maize from flowering to maturity. Sci. Agric. Sin..

[48] Song L., Liu D. (2015). Mutations in the three Arabidopsis genes that encode the E2 subunit of the mitochondrial pyruvate de-hydrogenase complex differentially affect enzymatic activity and plant growth. Plant Cell Rep..

[49] Liao J., Guan Y., Chen W., Shi C., Yao D., Wang F., Lam S.M., Shui G., Cao X. (2019). ACBD3 is required for FAPP2 trans-ferring glucosylceramide through maintaining the Golgi integrity. J. Mol. Cell Biol..

[50] Hsiao A.-S., Haslam R.P., Michaelson L.V., Liao P., Chen Q.-F., Sooriyaarachchi S., Mowbray S.L., Napier J.A., Tanner J.A., Chye M.-L. (2014). Arabidopsis cytosolic acyl-CoA-binding proteins ACBP4, ACBP5 and ACBP6 have overlapping but distinct roles in seed development. Biosci. Rep..

[51] Xie L.J., Yu L.J., Chen Q.F., Wang F.Z., Huang L., Xia F.N., Zhu T.R., Wu J.X., Yin J., Liao B. (2014). Arabidopsis acyl-CoA-binding protein ACBP3 participates in plant response to hypoxia by modulating very-long-chain fatty acid metabolism. Plant J..

[52] Tyagi V., Parihar V., Singh D., Kapoor S., Kapoor M. (2021). The DEAD-box RNA helicase eIF4A1 interacts with the SWI2/SNF2-related chromatin remodelling ATPase DDM1 in the moss Physcomitrella. Biochim. Biophys. Acta Proteins Proteom..

[53] Bradbury P.J., Zhang Z., Kroon D.E., Casstevens T.M., Ramdoss Y., Buckler E.S. (2007). TASSEL: Software for association mapping of complex traits in diverse samples. Bioinformatics.

[54] Yang H., Wang K. (2015). Genomic variant annotation and prioritization with ANNOVAR and wANNOVAR. Nat. Protoc..

[55] Price M.N., Dehal P.S., Arkin A.P. (2010). FastTree 2—Approximately Maximum-Likelihood Trees for Large Alignments. PLoS ONE.

[56] Yang J., Lee S.H., Goddard M.E., Visscher P.M. (2011). GCTA: A Tool for Genome-wide Complex Trait Analysis. Am. J. Hum. Genet..

[57] Alexander D.H., Novembre J., Lange K. (2009). Fast model-based estimation of ancestry in unrelated individuals. Genome Res..

[58] Zhang C., Dong S.-S., Xu J.-Y., He W.-M., Yang T.-L. (2019). PopLDdecay: A fast and effective tool for linkage disequilibrium decay analysis based on variant call format files. Bioinformatics.

[59] Danecek P., Auton A., Abecasis G., Albers C.A., Banks E., DePristo M.A., Handsaker R.E., Lunter G., Marth G.T., Sherry S.T. (2011). The variant call format and VCFtools. Bioinformatics.

[60] Du Z., Zhou X., Ling Y., Zhang Z., Su Z. (2010). agriGO: A GO analysis toolkit for the agricultural community. Nucleic Acids Res..

[61] Turner S.D. (2018). qqman: An R package for visualizing GWAS results using Q-Q and manhattan plots. J. Open Source Softw..

[62] Benjamini Y., Hochberg Y. (1995). Controlling the False Discovery Rate: A Practical and Powerful Approach to Multiple Testing. J. R. Stat. Soc. Ser. B.

[63] Livak K.J., Schmittgen T.D. (2001). Analysis of relative gene expression data using real-time quantitative PCR and the 2^−ΔΔCT^ method. Methods.

