# Supplementary figures and images for: Identification of Genetic Variations and Candidate Genes Responsible for Stalk Sugar Content and Agronomic Traits in Fresh Corn via GWAS across Multiple Environments
Source: Int J Mol Sci. 2022 Nov 4;23(21):13490. doi: 10.3390/ijms232113490 (PMC9655584; doi:10.3390/ijms232113490)

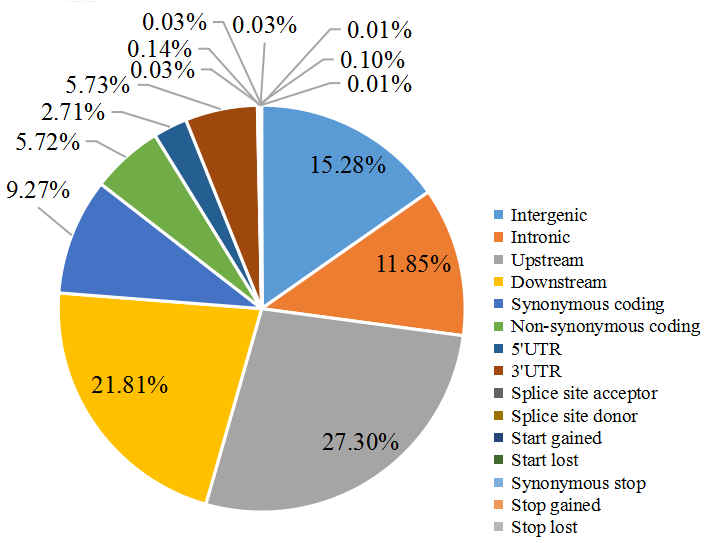

Supplement: Supplementary file 1 [file ijms-23-13490-s001.zip › Figure S1.tif]

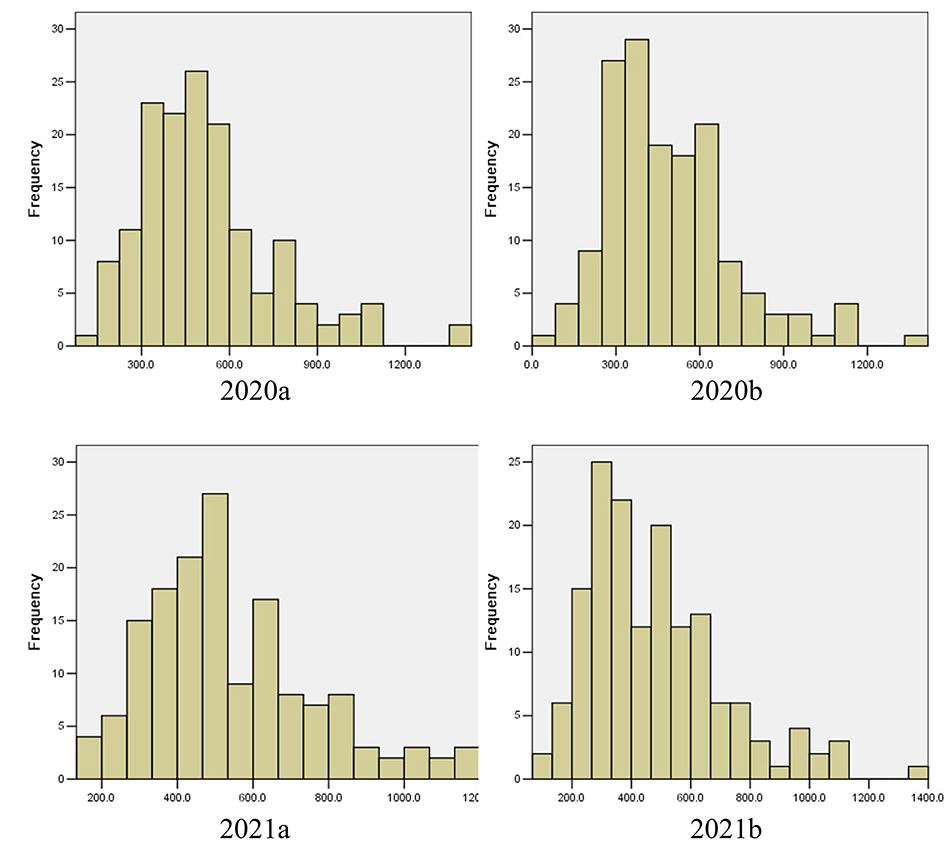

Supplement: Supplementary file 1 [file ijms-23-13490-s001.zip › Figure S10.tif]

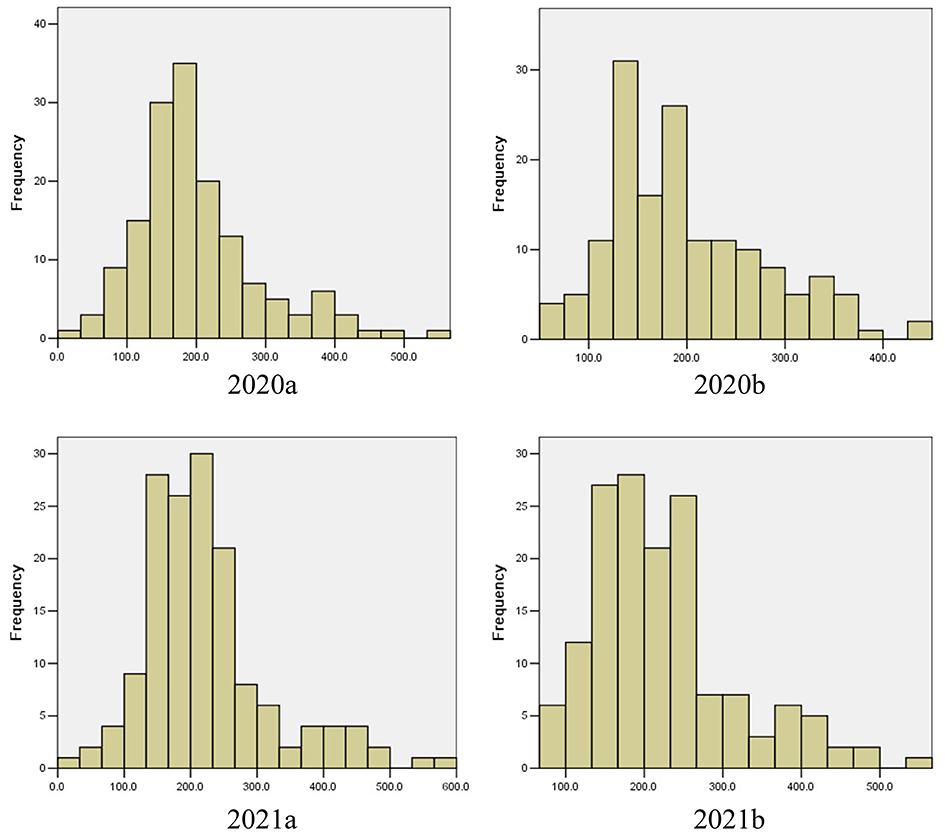

Supplement: Supplementary file 1 [file ijms-23-13490-s001.zip › Figure S11.tif]

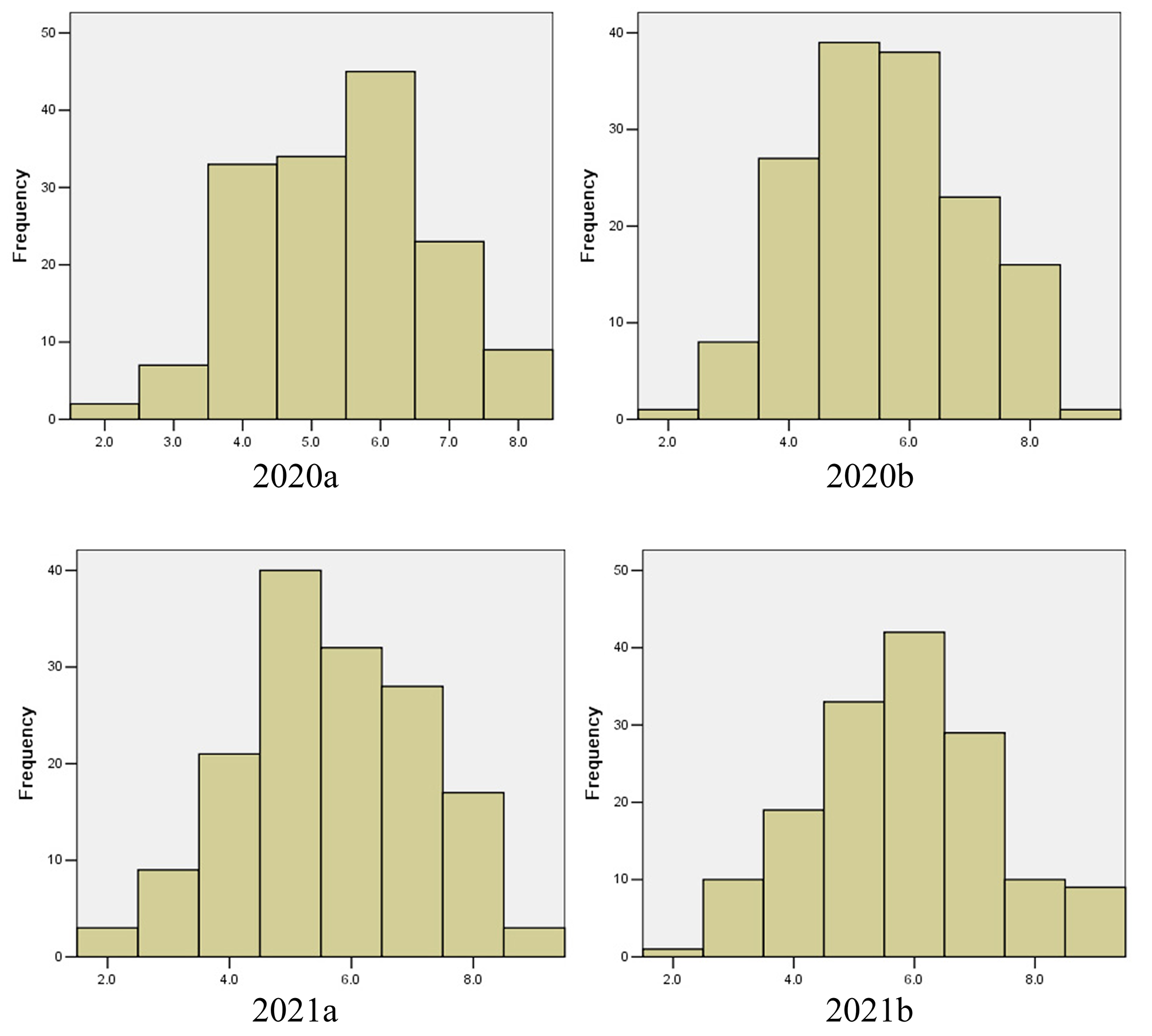

Supplement: Supplementary file 1 [file ijms-23-13490-s001.zip › Figure S12.tif]

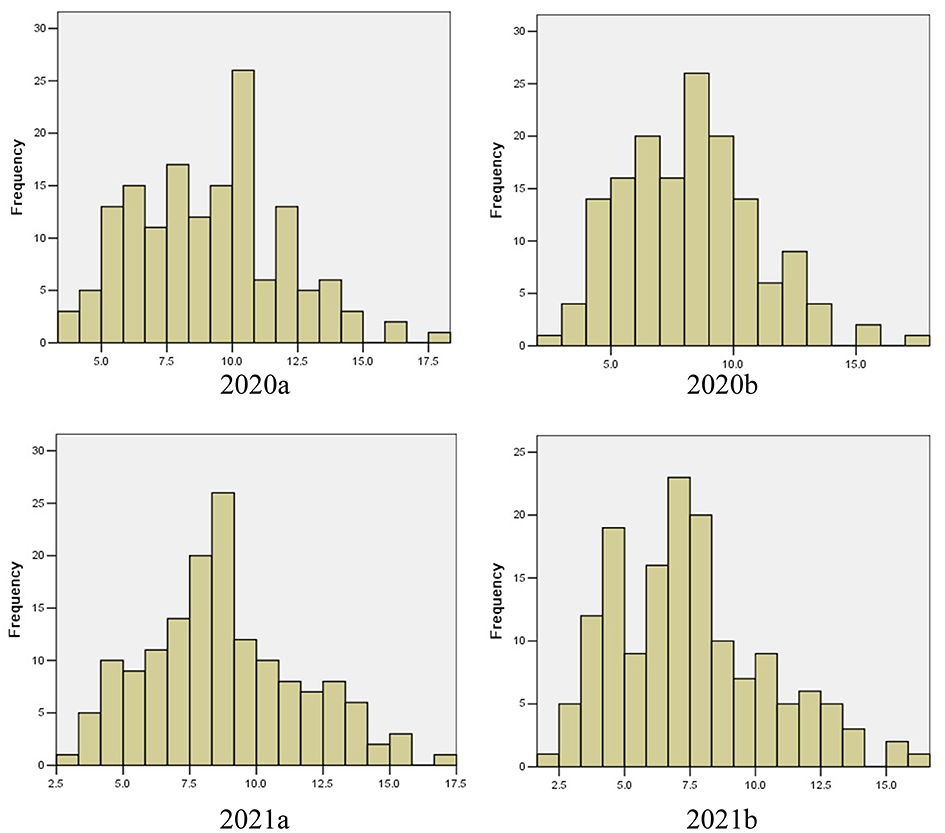

Supplement: Supplementary file 1 [file ijms-23-13490-s001.zip › Figure S13.tif]

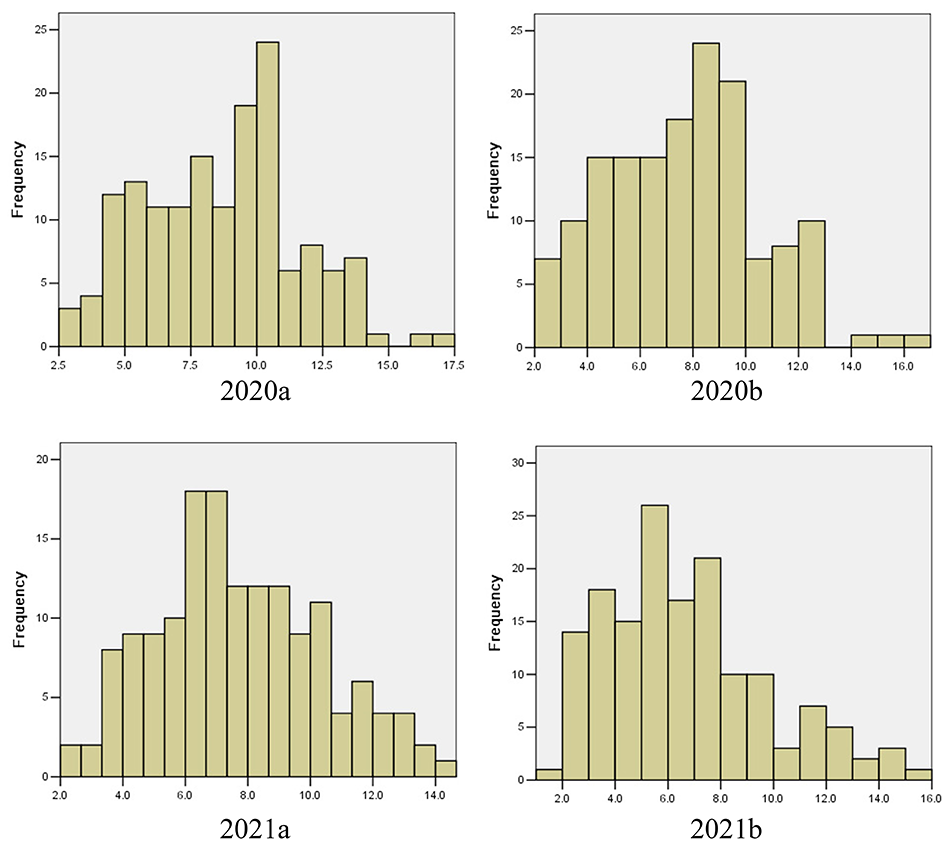

Supplement: Supplementary file 1 [file ijms-23-13490-s001.zip › Figure S14.tif]

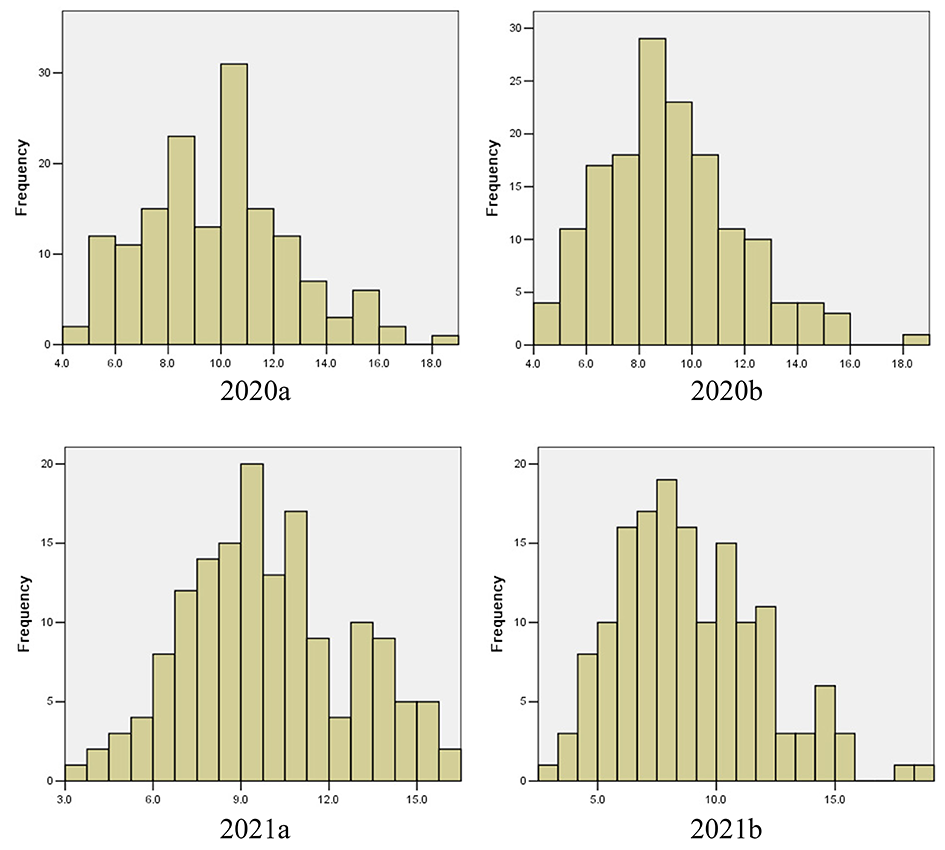

Supplement: Supplementary file 1 [file ijms-23-13490-s001.zip › Figure S15.tif]

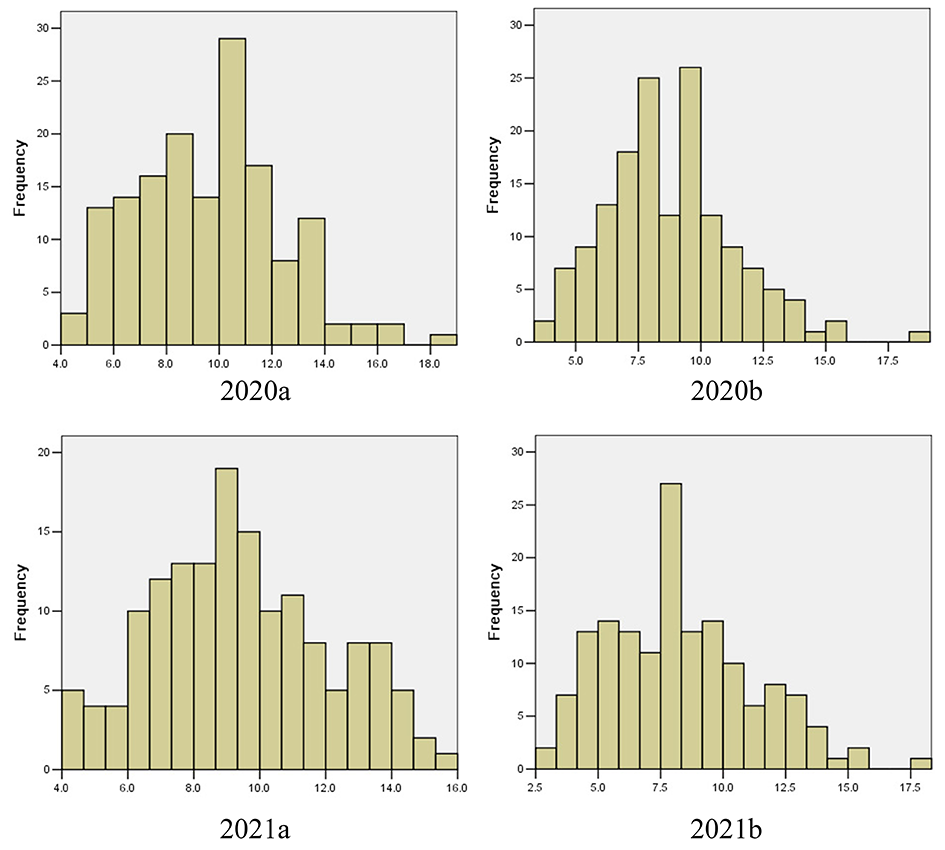

Supplement: Supplementary file 1 [file ijms-23-13490-s001.zip › Figure S16.tif]

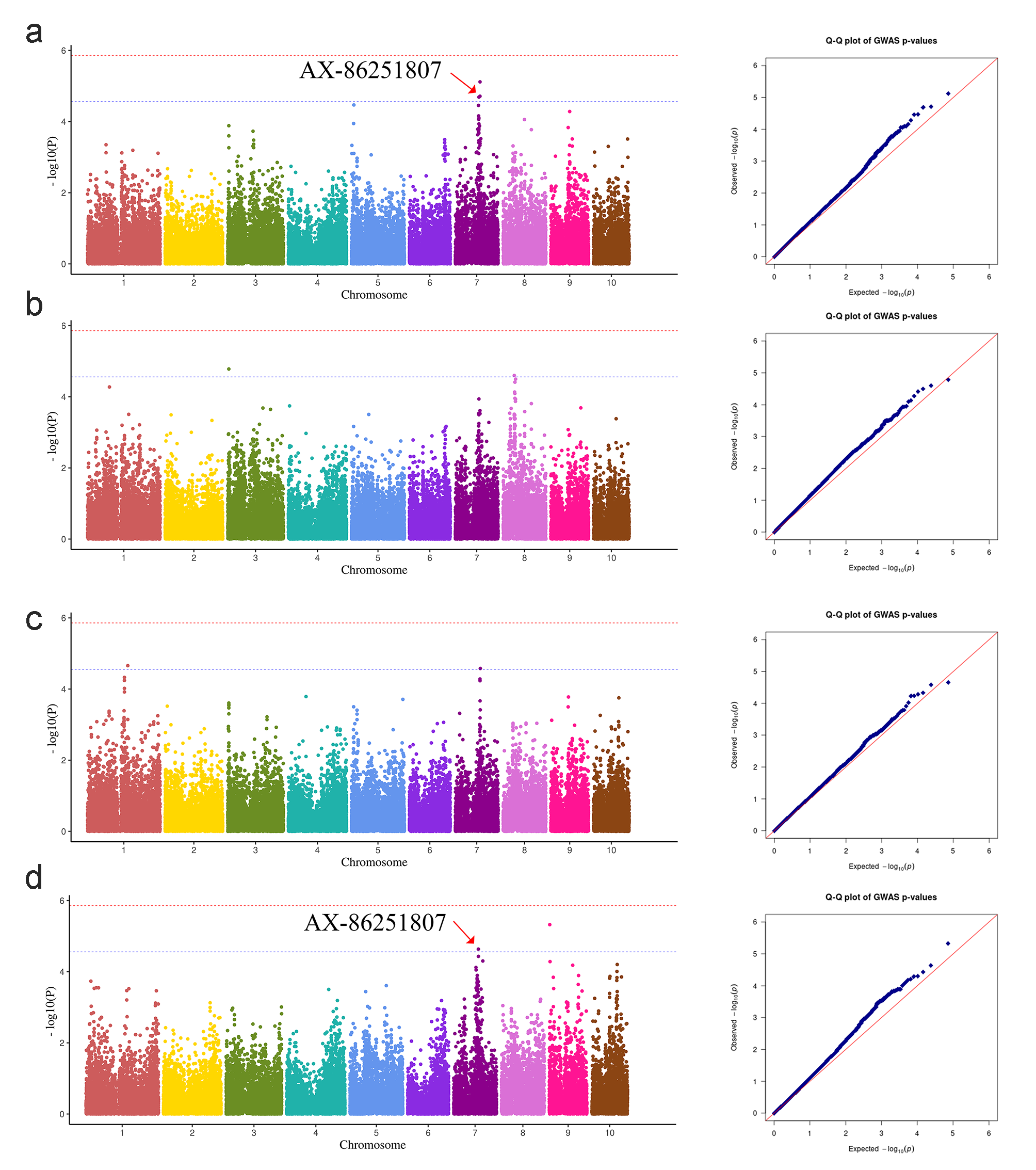

Supplement: Supplementary file 1 [file ijms-23-13490-s001.zip › Figure S17.tif]

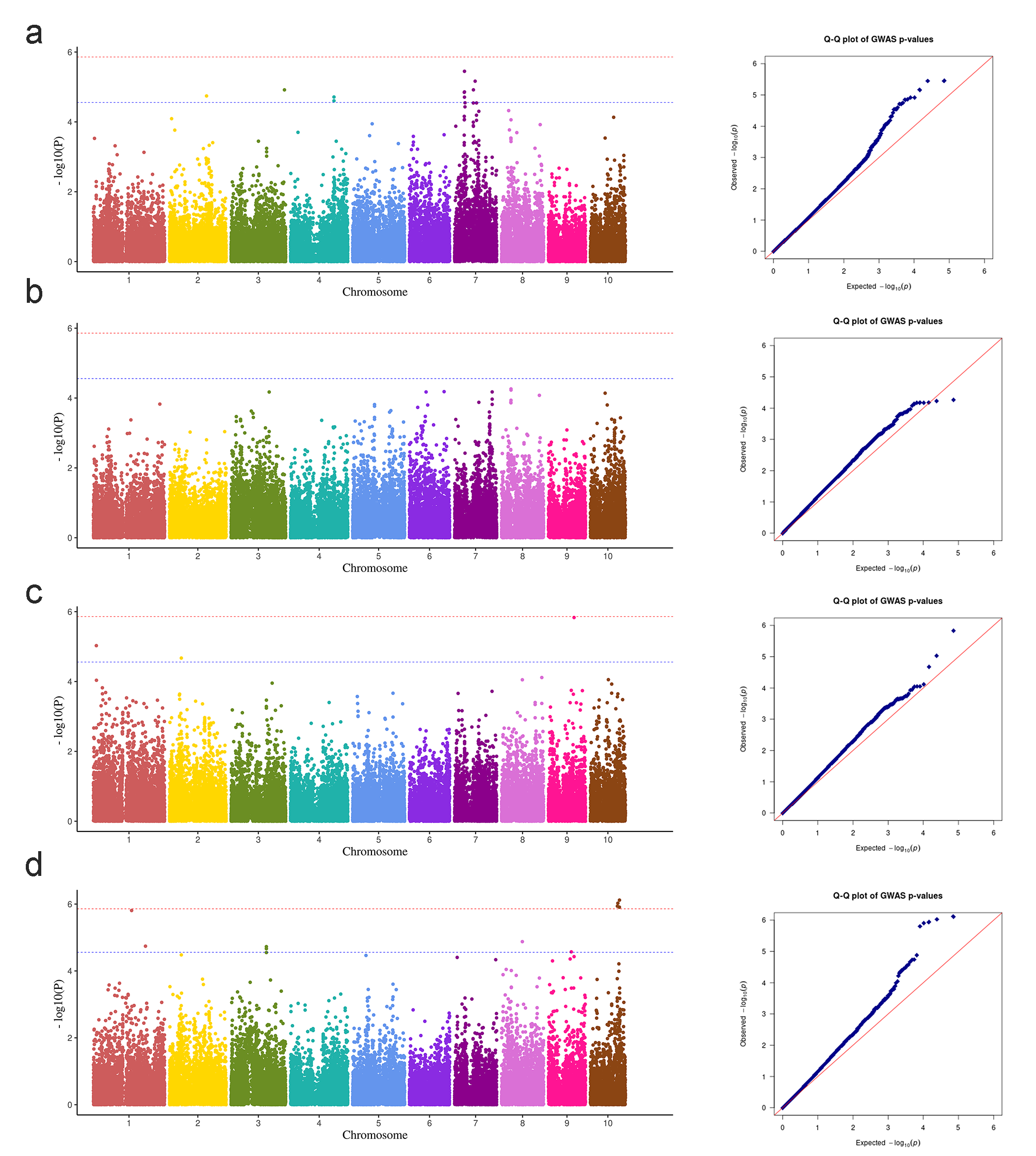

Supplement: Supplementary file 1 [file ijms-23-13490-s001.zip › Figure S18.tif]

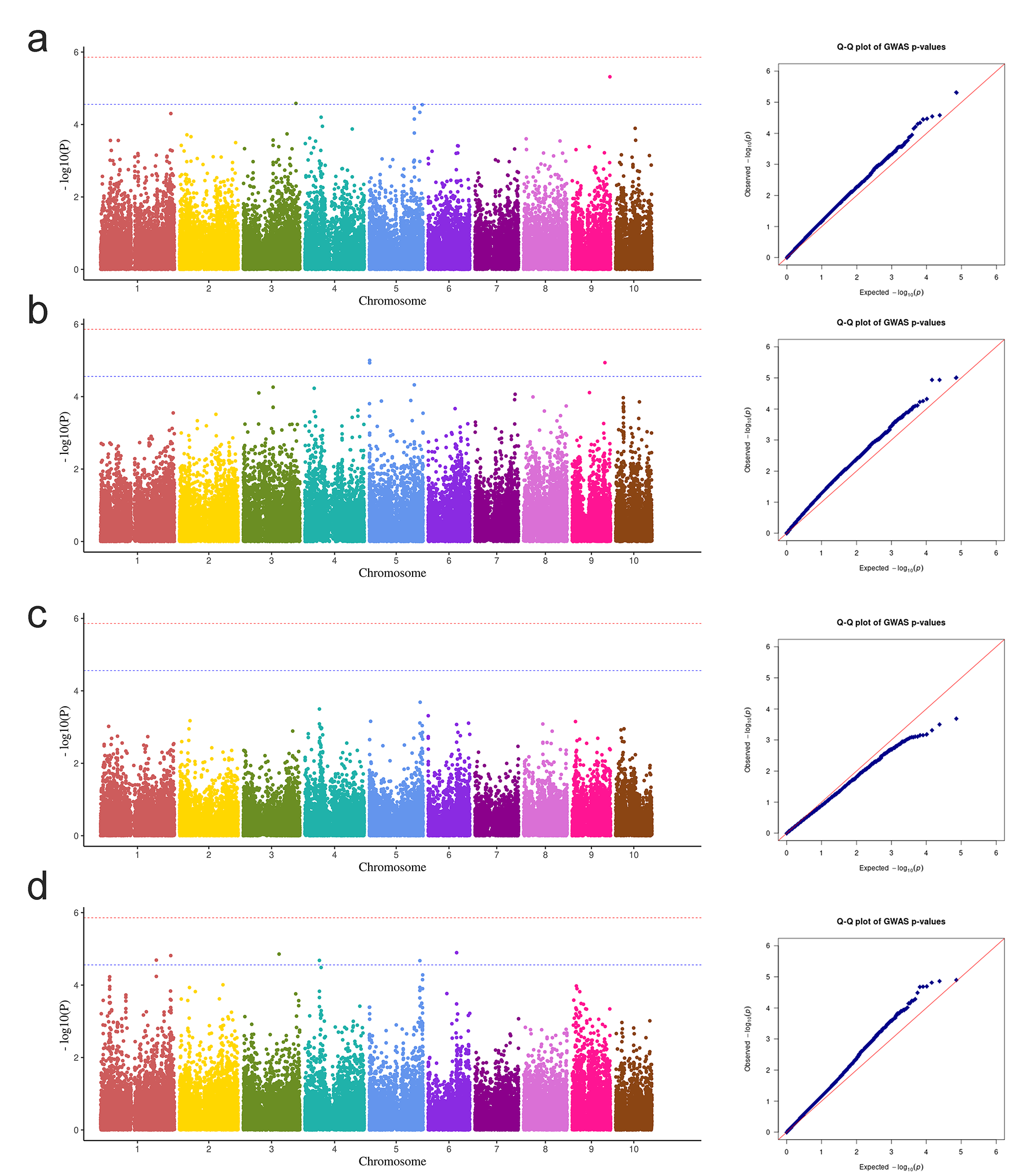

Supplement: Supplementary file 1 [file ijms-23-13490-s001.zip › Figure S19.tif]

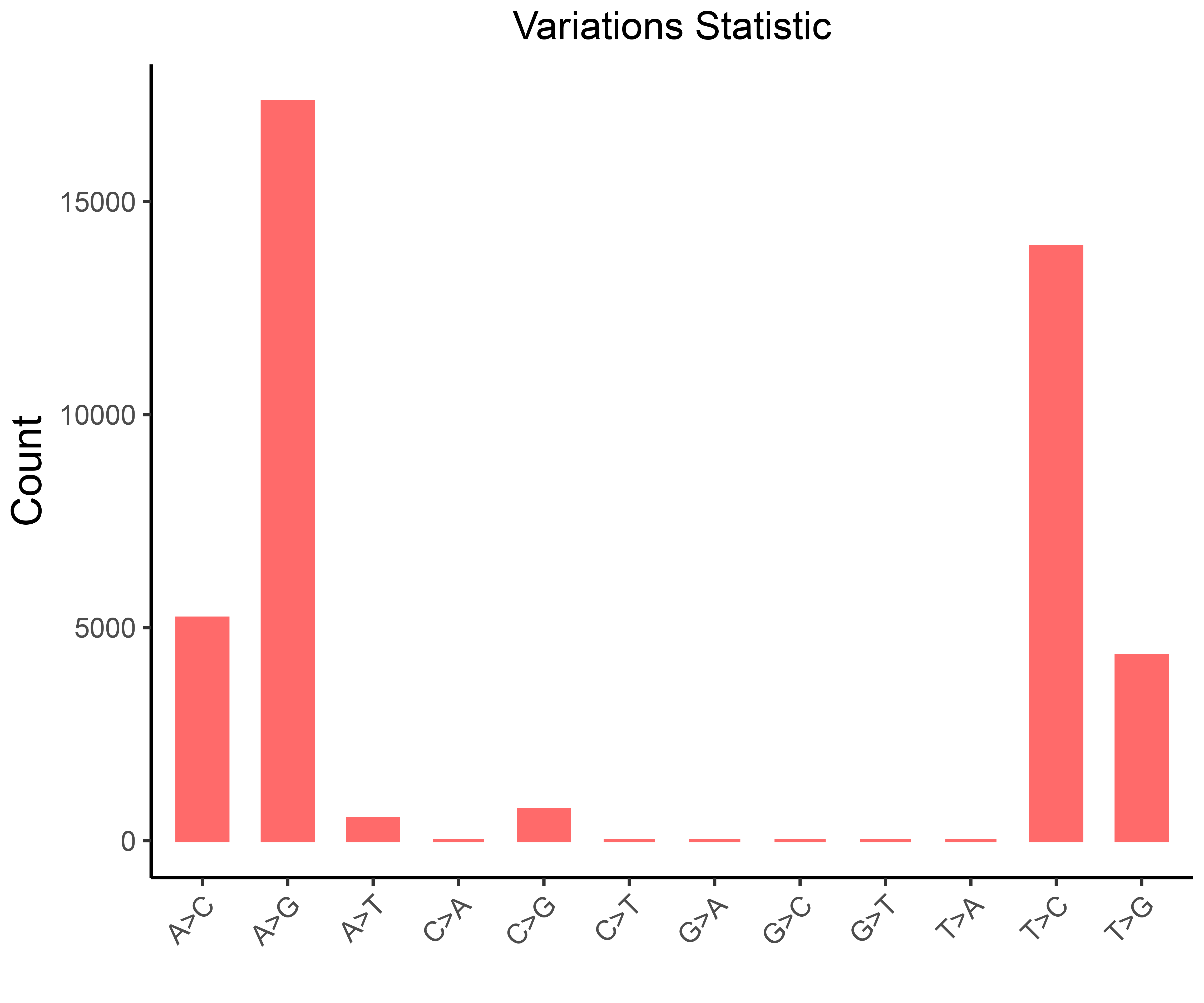

Supplement: Supplementary file 1 [file ijms-23-13490-s001.zip › Figure S2.tif]

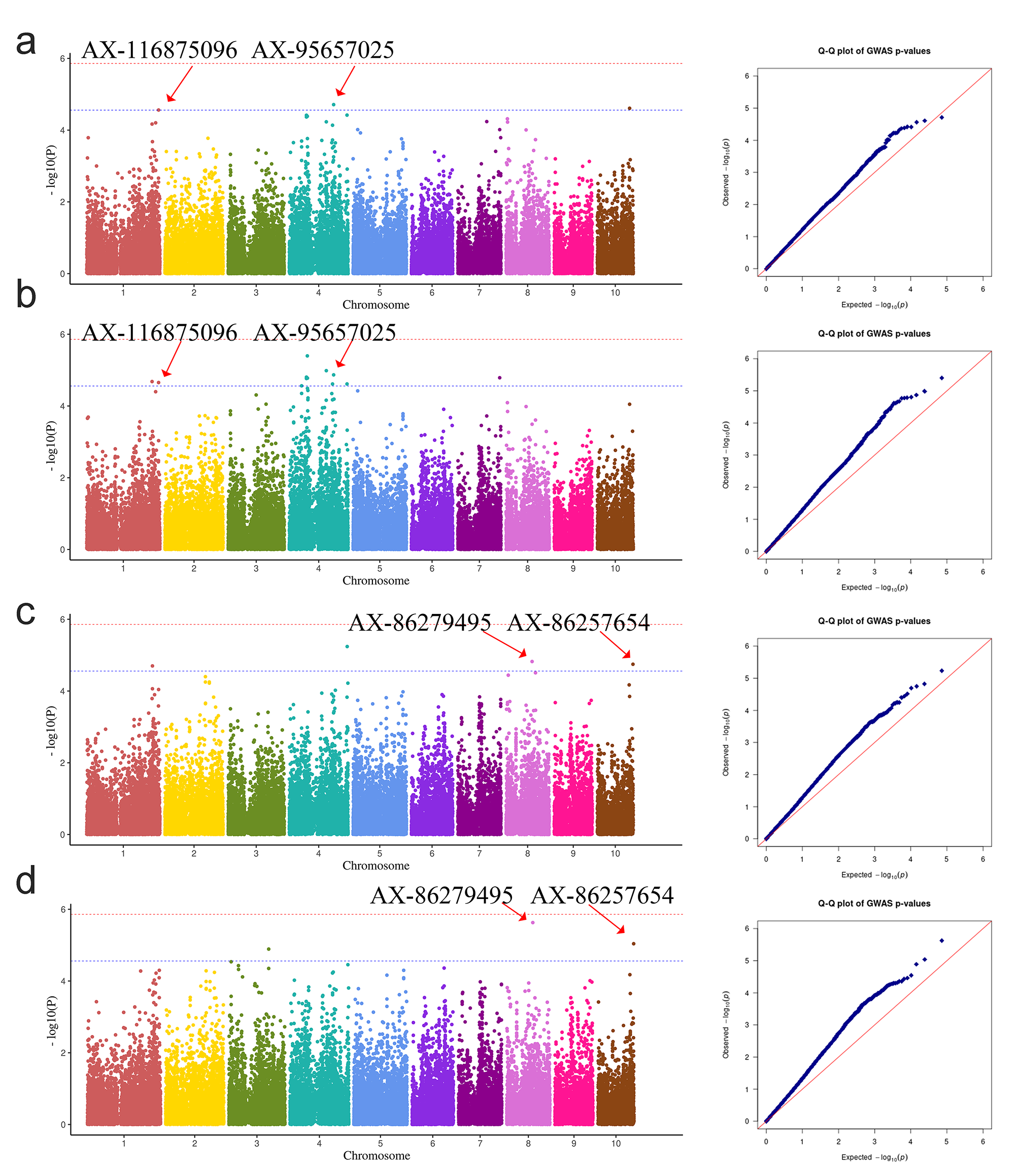

Supplement: Supplementary file 1 [file ijms-23-13490-s001.zip › Figure S20.tif]

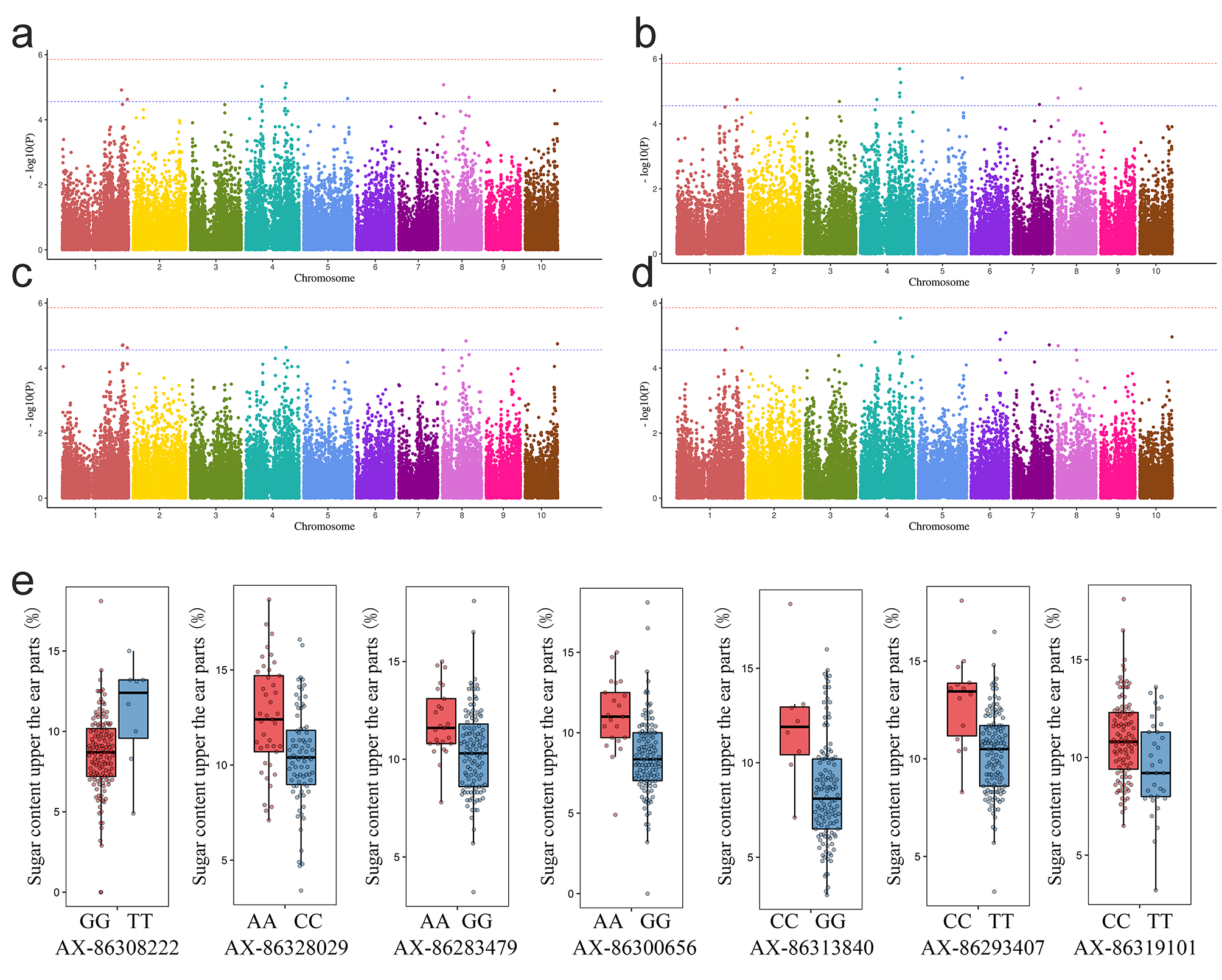

Supplement: Supplementary file 1 [file ijms-23-13490-s001.zip › Figure S21.tif]

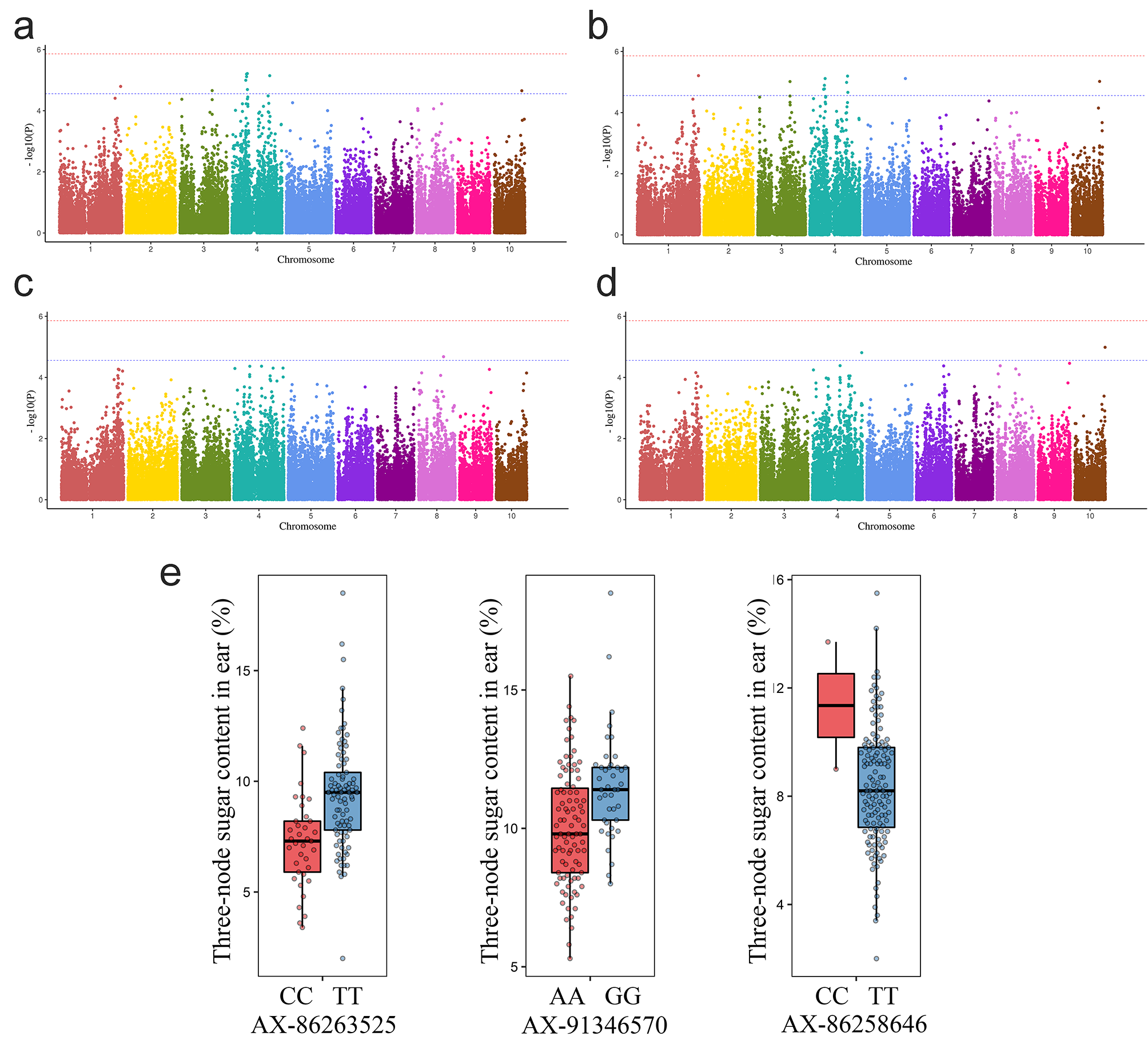

Supplement: Supplementary file 1 [file ijms-23-13490-s001.zip › Figure S22.tif]

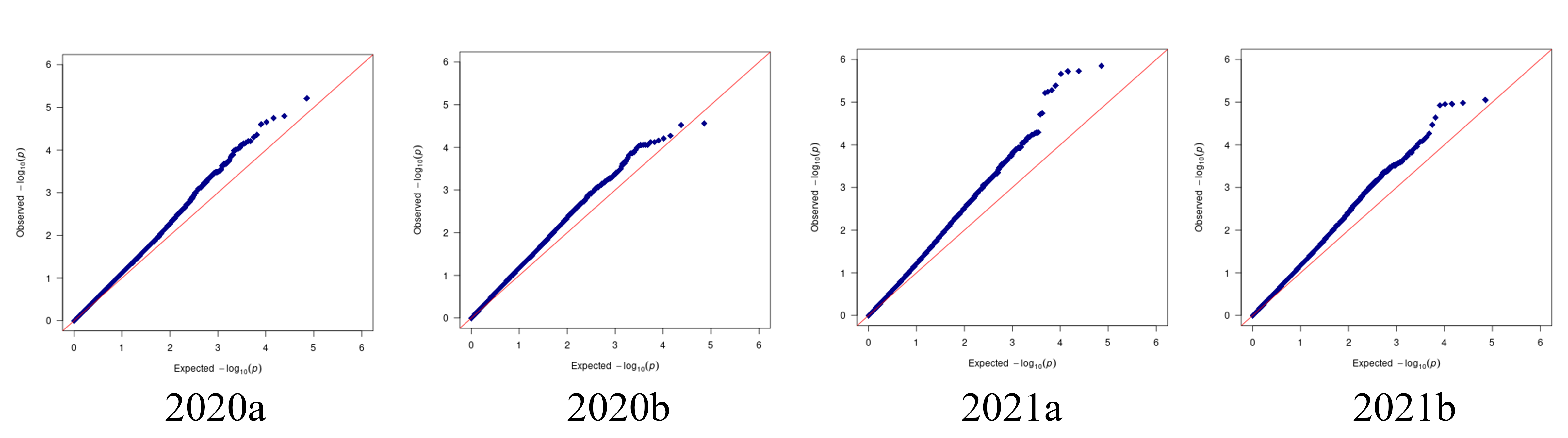

Supplement: Supplementary file 1 [file ijms-23-13490-s001.zip › Figure S23.tif]

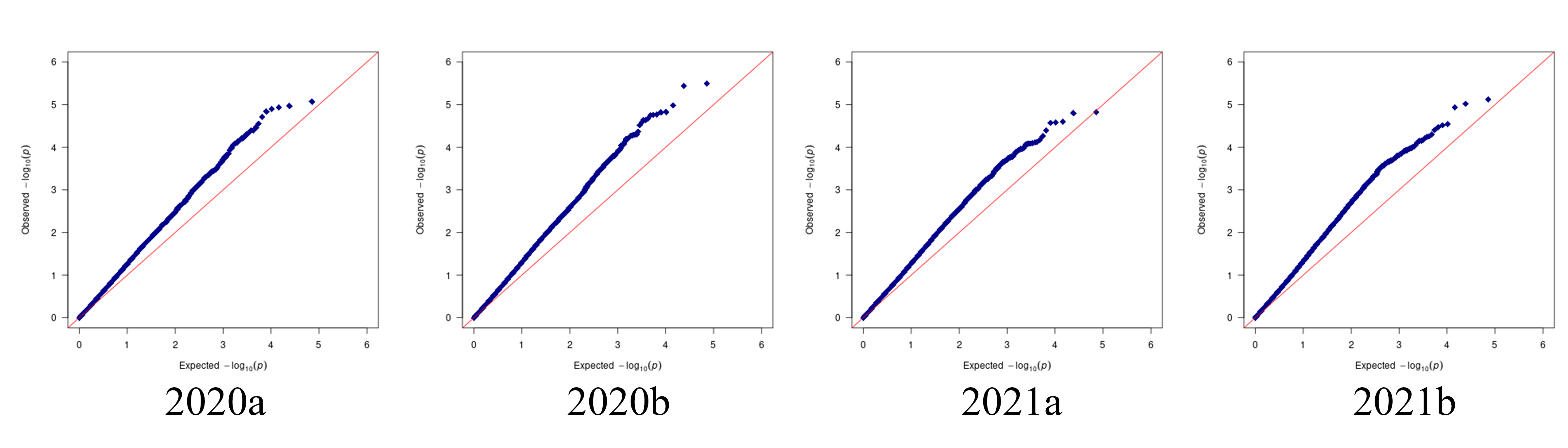

Supplement: Supplementary file 1 [file ijms-23-13490-s001.zip › Figure S24.tif]

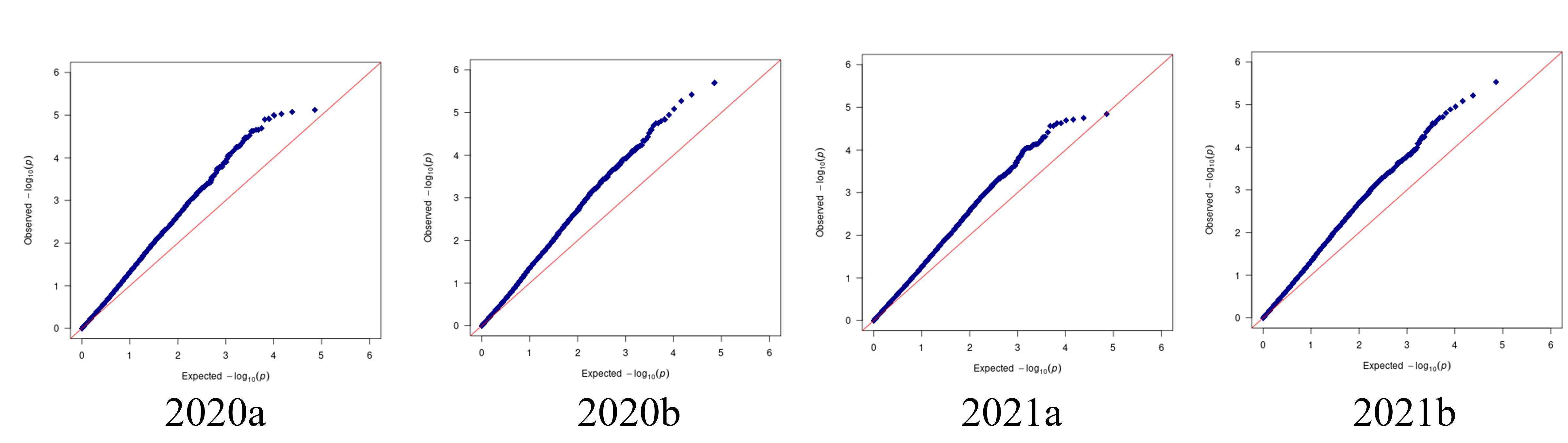

Supplement: Supplementary file 1 [file ijms-23-13490-s001.zip › Figure S25.tif]

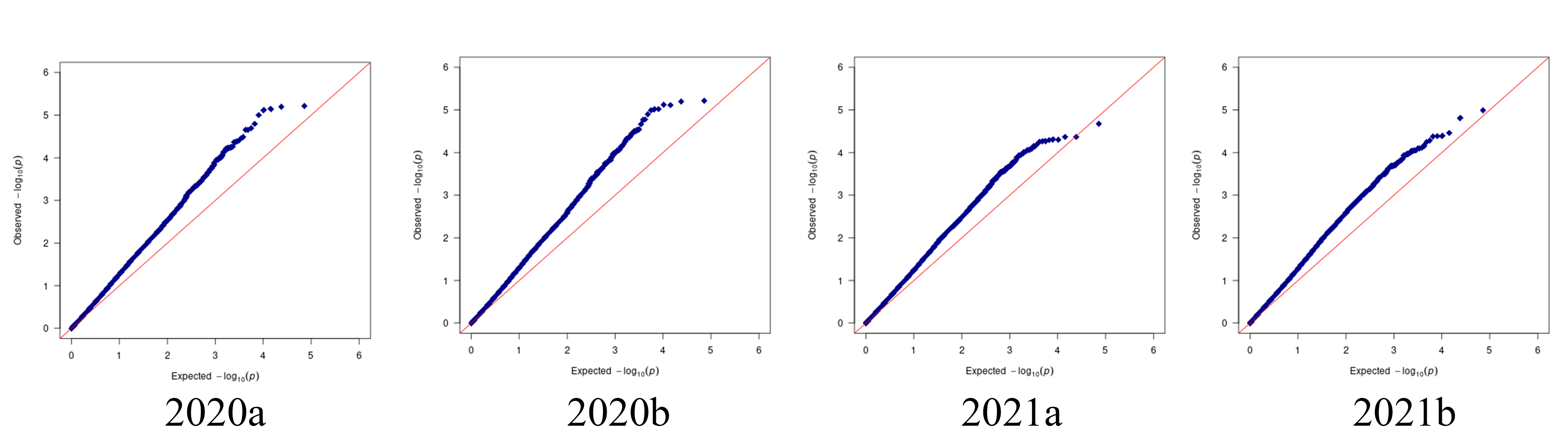

Supplement: Supplementary file 1 [file ijms-23-13490-s001.zip › Figure S26.tif]

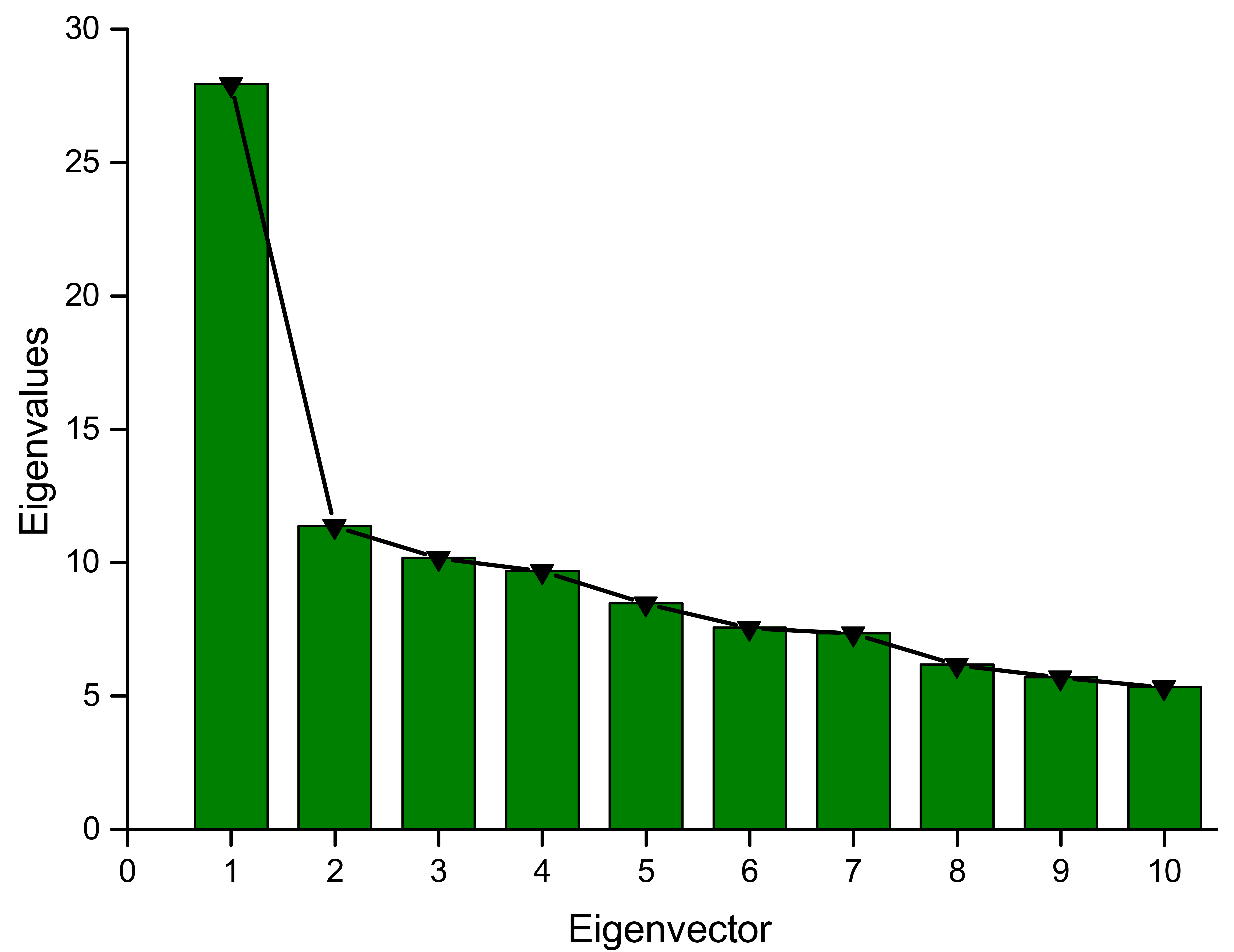

Supplement: Supplementary file 1 [file ijms-23-13490-s001.zip › Figure S3.tif]

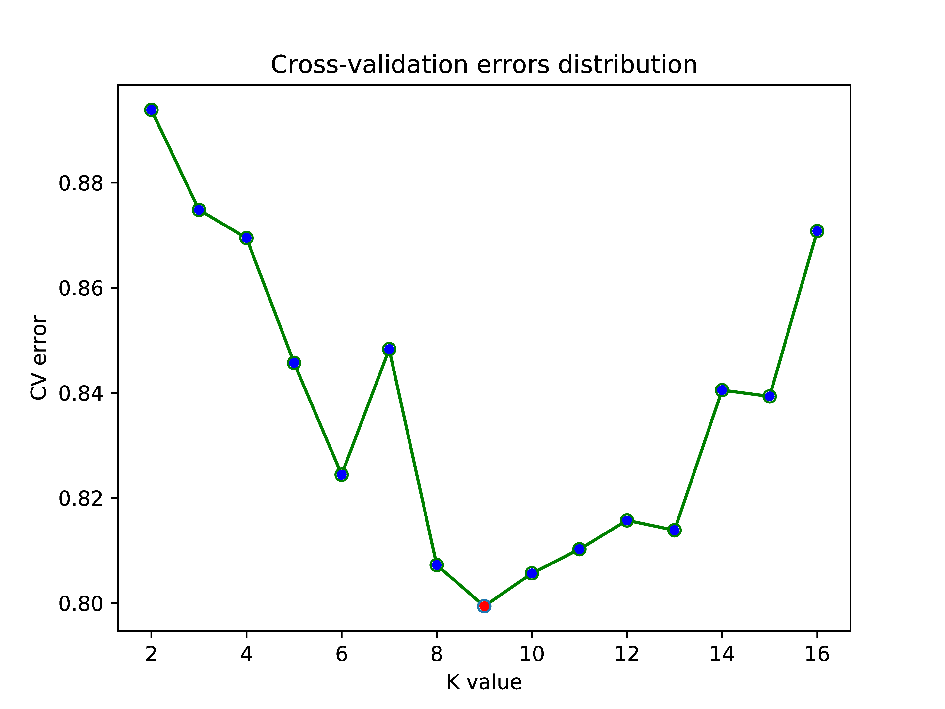

Supplement: Supplementary file 1 [file ijms-23-13490-s001.zip › Figure S4.png]

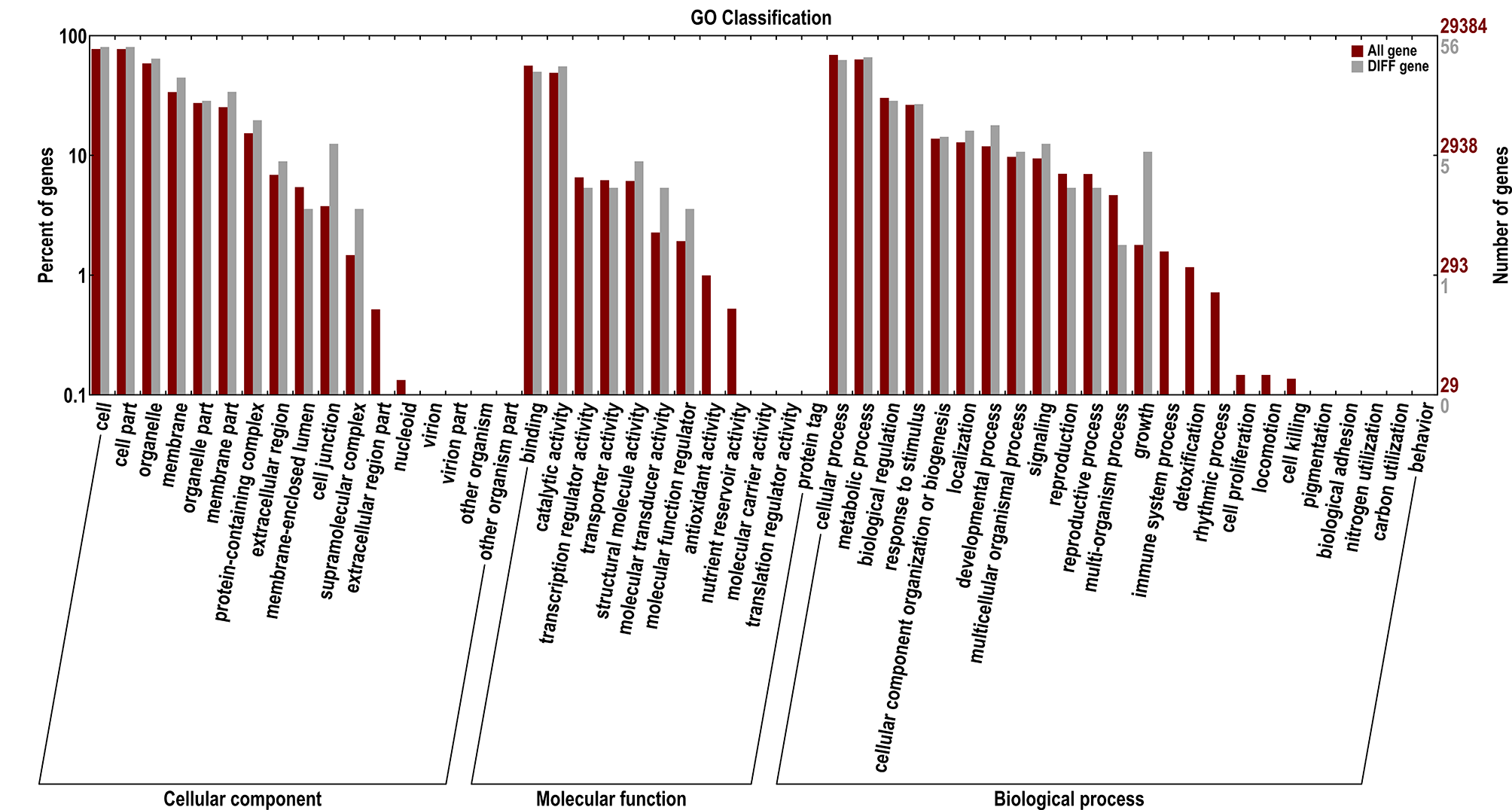

Supplement: Supplementary file 1 [file ijms-23-13490-s001.zip › Figure S5.tif]

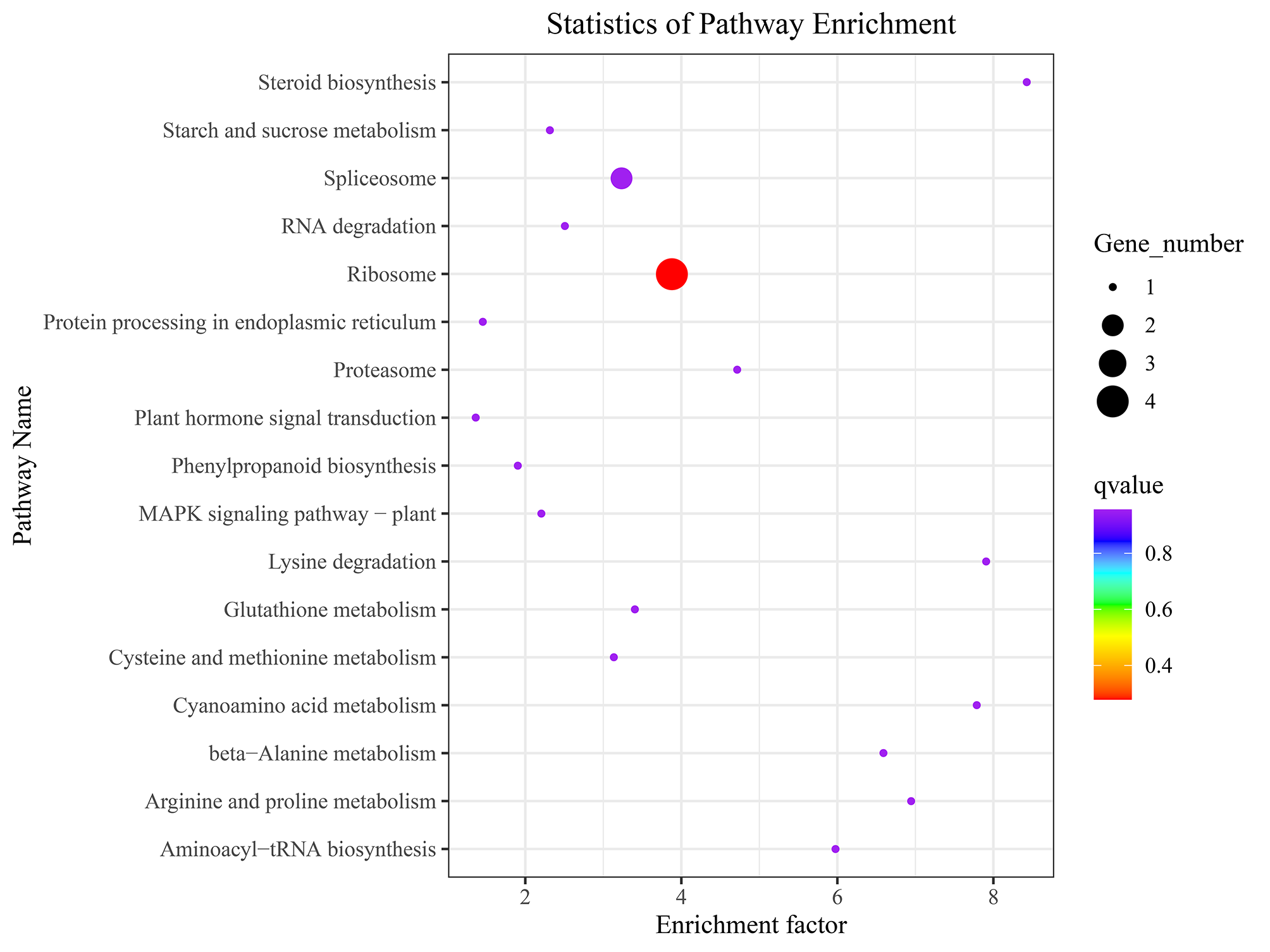

Supplement: Supplementary file 1 [file ijms-23-13490-s001.zip › Figure S6.tif]

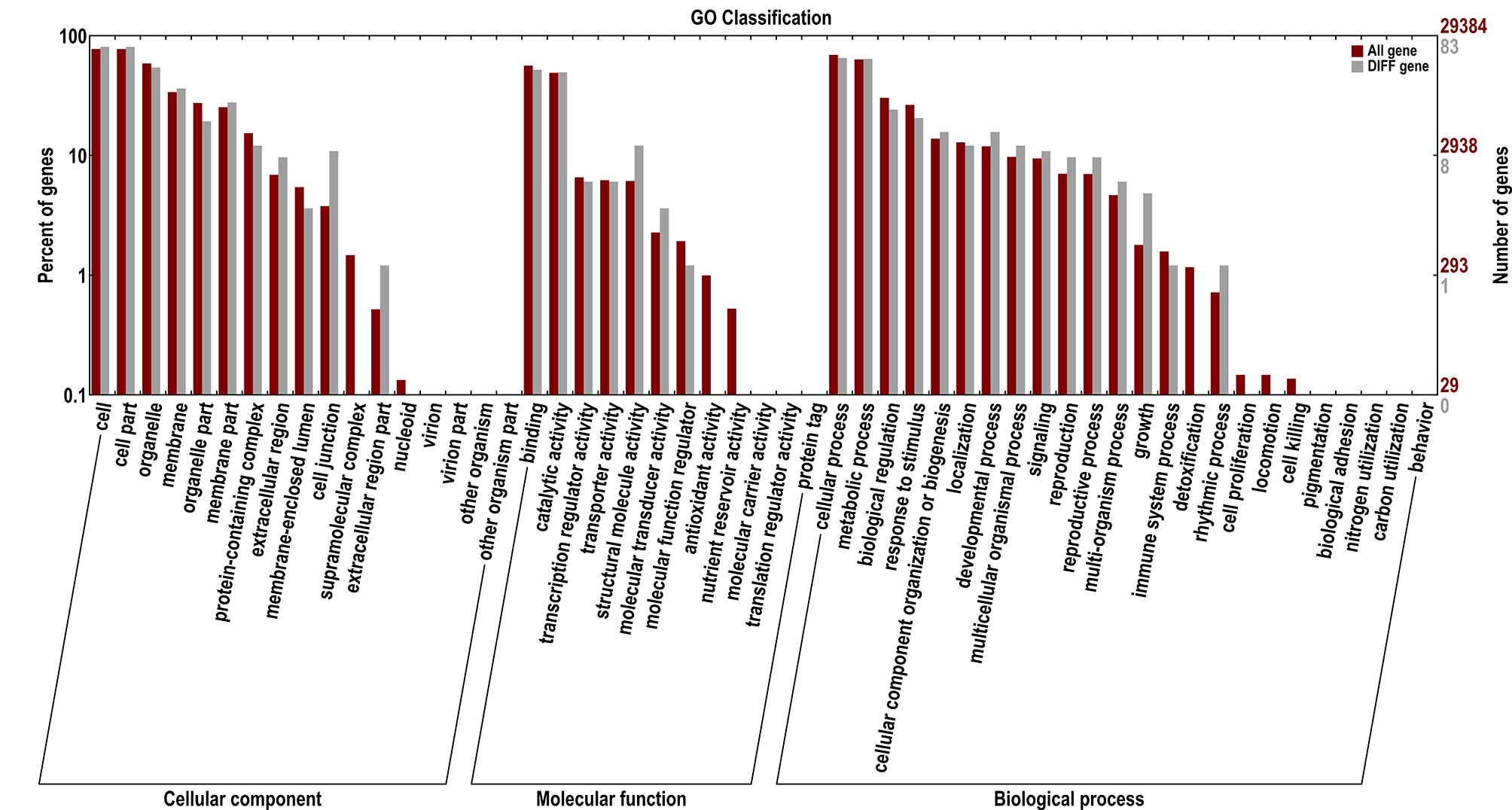

Supplement: Supplementary file 1 [file ijms-23-13490-s001.zip › Figure S7.tif]

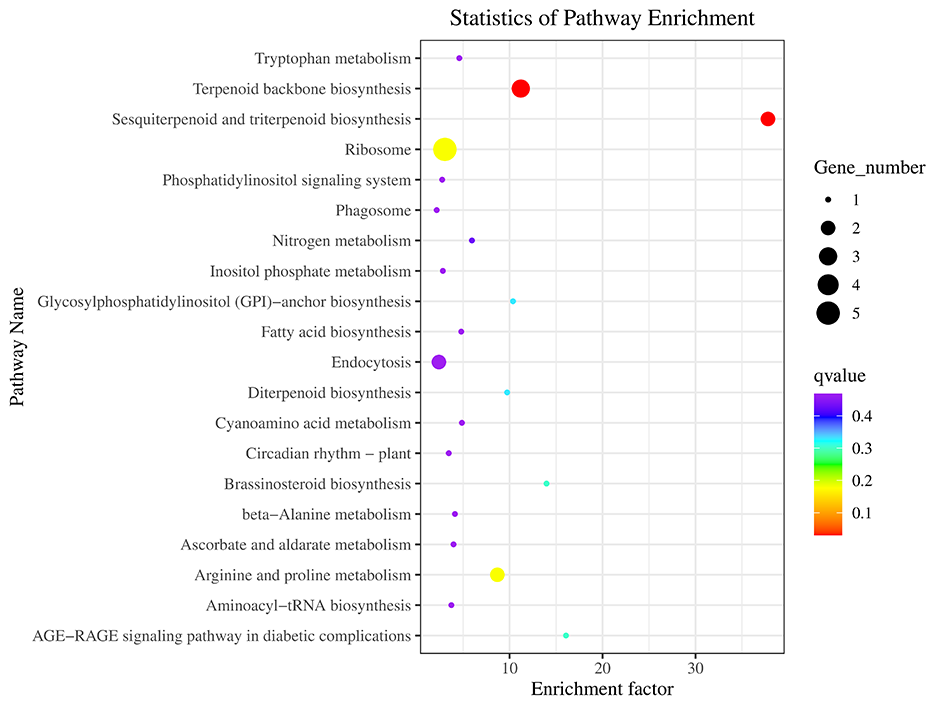

Supplement: Supplementary file 1 [file ijms-23-13490-s001.zip › Figure S8.tif]

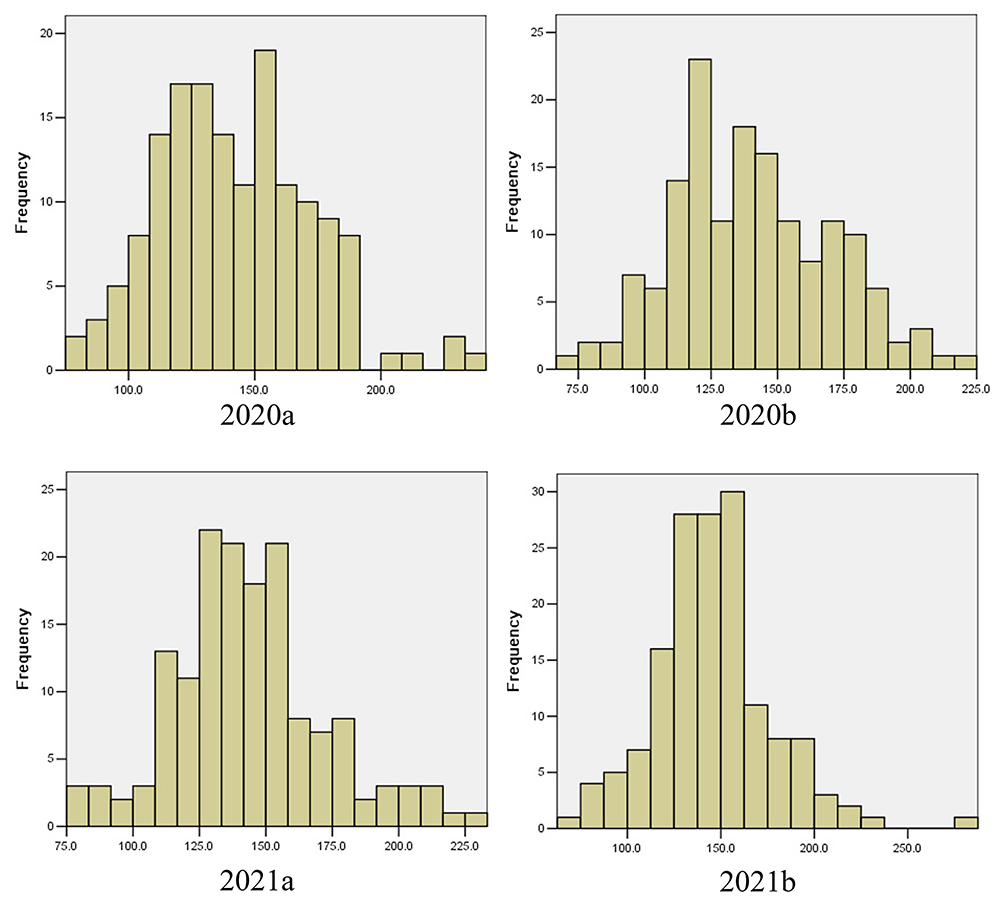

Supplement: Supplementary file 1 [file ijms-23-13490-s001.zip › Figure S9.tif]
